# Microbiome signature and diversity regulates the level of energy production under anaerobic condition

**DOI:** 10.1038/s41598-021-99104-3

**Published:** 2021-10-05

**Authors:** M. Shaminur Rahman, M. Nazmul Hoque, Joynob Akter Puspo, M. Rafiul Islam, Niloy Das, Mohammad Anwar Siddique, M. Anwar Hossain, Munawar Sultana

**Affiliations:** 1 Department of Microbiology, University of Dhaka, Dhaka, 1000 Bangladesh; 2 Department of Gynecology, Obstetrics and Reproductive Health, Bangabandhu Sheikh Mujibur Rahman Agricultural University, Gazipur, 1706 Bangladesh; 3 Surge Engineering (www.surgeengineering.com), Dhaka, 1205 Bangladesh; 4 Present Address: Jashore University of Science and Technology, Jashore, 7408 Bangladesh

**Keywords:** Biotechnology, Computational biology and bioinformatics, Microbiology

## Abstract

The microbiome of the anaerobic digester (AD) regulates the level of energy production. To assess the microbiome diversity and composition in different stages of anaerobic digestion, we collected 16 samples from the AD of cow dung (CD) origin. The samples were categorized into four groups (Group-I, Group-II, Group-III and Group-IV) based on the level of energy production (CH_4_%), and sequenced through whole metagenome sequencing (WMS). Group-I (n = 2) belonged to initial time of energy production whereas Group-II (n = 5), Group-III (n = 5), and Group-IV (n = 4) had 21–34%, 47–58% and 71–74% of CH_4_, respectively. The physicochemical analysis revealed that level of energy production (CH_4_%) had significant positive correlation with digester pH (r = 0.92, p < 0.001), O_2_ level (%) (r = 0.54, p < 0.05), and environmental temperature (°C) (r = 0.57, p < 0.05). The WMS data mapped to 2800 distinct bacterial, archaeal and viral genomes through PathoScope (PS) and MG-RAST (MR) analyses. We detected 768, 1421, 1819 and 1774 bacterial strains in Group-I, Group-II, Group-III and Group-IV, respectively through PS analysis which were represented by *Firmicutes*, *Bacteroidetes*, *Proteobacteria*, *Actinobacteria*, *Spirochaetes* and *Fibrobacteres* phyla (> 93.0% of the total abundances). Simultaneously, 343 archaeal strains were detected, of which 95.90% strains shared across four metagenomes. We identified 43 dominant species including 31 bacterial and 12 archaeal species in AD microbiomes, of which only archaea showed positive correlation with digester pH, CH_4_ concentration, pressure and temperature (Spearman correlation; r > 0.6, p < 0.01). The indicator species analysis showed that the species *Methanosarcina vacuolate*, *Dehalococcoides mccartyi*, *Methanosarcina* sp. Kolksee and *Methanosarcina barkeri* were highly specific for energy production. The correlation network analysis showed that different strains of *Euryarcheota* and *Firmicutes* phyla exhibited significant correlation (p = 0.021, Kruskal–Wallis test; with a cutoff of 1.0) with the highest level (74.1%) of energy production (Group-IV). In addition, top CH_4_ producing microbiomes showed increased genomic functional activities related to one carbon and biotin metabolism, oxidative stress, proteolytic pathways, membrane-type-1-matrix-metalloproteinase (MT1-MMP) pericellular network, acetyl-CoA production, motility and chemotaxis. Importantly, the physicochemical properties of the AD including pH, CH_4_ concentration (%), pressure, temperature and environmental temperature were found to be positively correlated with these genomic functional potentials and distribution of ARGs and metal resistance pathways (Spearman correlation; r > 0.5, p < 0.01). This study reveals distinct changes in composition and diversity of the AD microbiomes including different indicator species, and their genomic features that are highly specific for energy production.

## Introduction

Bangladesh is experiencing rapidly increased energy consumption over the past two decades. Being one of the world’s most densely populated and least urbanized countries, around 72% of population of this country live in rural areas where there is no supply of natural gas, the main source of energy^1^. The access to clean and affordable energy is one of the prerequisites to achieve the sustainable development in rural areas. Bangladesh is currently endowed with 25.7 million cattle, 0.83 million buffaloes, 14.8 million goats, 1.9 million sheep, 118.7 million chicken and 34.1 million ducks, and the manure from these animals could provide a reliable source of clean energy^2^. The production of biogas from CD through anaerobic digestion process cannot only provide fuel, but is also important for reduction of fertilizer nutrient utilization and rural forest conservation in rural areas^3,4^. The fecal materials of cattle or CD harbors a rich microbial diversity, containing different species of bacteria (*Bacillus* spp., *Corynebacterium* spp. *Citrobacter koseri*, *Enterobacter aerogenes*, *Escherichia coli*, *Klebsiella oxytoca*, *K. pneumoniae*, *Kluyvera* spp., *Morgarella morganii*, *Pasteurella* spp., *Providencia alcaligenes*, *Providencia stuartii*, *Pseudomonas* spp., and *Lactobacillus* spp.), protozoa and yeast (*Saccharomyces* and *Candida*), which makes them suitable for microbial degradation of wastes^5,6^. The slurry from CD was maintained in the ratio of 1:10 or 1:25 is able to degrade the rural, urban and hospital wastes, including oil spillage to five basic elements^5,6^. Recent research findings indicate that CD can supply nutrients and energy required for microbial growth thereby resulting in the bioremediation of pollutants, and energy production^5,7^.

Biogas (CH_4_), produced from an AD is an emerging renewable source of energy. This is a relatively high-value fuel and continuous source of energy supply to solve the environmental and energy challenges to replace natural gas or transportation fuel, and an insurance for future energy in a sustainable and environment-friendly manner^8^. Anaerobic environments play critical roles in the global carbon cycle through the digestion of organic agricultural waste, manure, municipal waste, digester materials, sewage, green waste or food. Moreover, anaerobic digestion of livestock manure improves organic fertilizer quality compared with undigested manure^9^, and the load of the pathogenic microorganisms and related antimicrobial resistance is also decreased through the biological process of anaerobic degradation^10^. The rising energy prices and increasing concern of emission of greenhouse gases are the major concern for the people and agro-industries worldwide to consider the wider application of AD technology. This sustainable technology has been viewed as a way to address environmental concern through the generation of CH_4_ within engineered bioreactors, and thereby reducing the human dependence on fossil fuels^11,12^. Furthermore, environment friendly renewable energy produced from locally available raw materials and recycled waste could thus contribute to climate change mitigation^8^. The renewable energy (CH_4_) produced from AD which is independent of weather conditions could serve for the production of electricity, heat and fuels^13^. Anaerobic transformation of organic wastes in the AD is carried out by different bacterial and archaeal species, such as hydrolytic, acid forming, acetogenic, and methanogens which produce CO_2_ and CH_4_ as the main products of the digestion process^11^. Though biogas production is directly influenced by the composition of the AD microbiomes^11,14^, the genomic potentials of the microbiomes favoring anaerobic metabolism to control the level of energy production is thermodynamically dependent on environmental parameters of the AD^15^.

Diverse microbial communities are associated with biomass decomposition and CH_4_ production through the metabolic activities of substrate hydrolysis, acidogenesis, acetogenesis and methanogenesis^16^. A clear understanding of the structure, composition and diversity of the multifarious microbial community involved in biogas production is crucial for the optimization of their performance and stable operational process of the AD. Moreover, a detailed insight into relevant microbial metabolic pathways involved in CH_4_ synthesis and syntropy is essential to upsurge the yield of biogas. However till to date, only few studies have focused on the taxonomic and functional characterization of microbiomes originating from both laboratory-scale^11^ and full-scale^11,17^ biogas reactors under different prevailing ecological conditions^14,18^. The conventional culture-based techniques^19,20^ for characterization of the microbiotas in different niches including controlled anaerobic chambers^13,21^ has been replaced during the last decade by the rapid advances in high-throughput NGS technology and bioinformatics tools^22,23^. Despite, the 16S rRNA partial gene sequencing approach remained the most widely used genomic approach to study the microbiomes of the AD^24,25^, several inherent limitations including the polymerase chain reaction (PCR) bias, inability to detect viruses, lower taxonomic resolution (up to genus level only), and limiting information on gene abundance and functional profiling have made this technique questionable^22,23^. Conversely, the shotgun WMS approach which can identify the total microbial components of a sample (including viruses, bacteria, archaea, fungi, and protists), is being used prudently to decipher the phylogenetic composition, microbiome structure and diversity including profiling of their functional characteristics and interconnections^24,26^. The current accelerated pace of genomic approaches identified more than 150 species of microorganisms^27^ such as *Clostridium bornimense*, *Herbinix hemicellulosilytica*, *Herbinix luporum*, *Herbivorax saccincola*, *Proteiniphilum saccharofermentans*, *Petrimonas mucosa*, *Fermentimonas caenicola*, and *Proteiniborus indolifex* or even their genomic features to be associated with increased production of biogas^11,14,17,27–29^. To address the changes in microbiome diversity and composition associated with different level of CH_4_ production, we present a comprehensive deep metagenomic (WMS) analysis of sixteen (n = 16) samples collected from AD periodically loaded with CD under different pH, CO_2_, O_2_, H_2_S and temperature level. Using a homogeneous mapping and annotation workflow associated with a de-replication strategy, our analyses identified ~ 2800 distinct bacterial and archaeal species along with their co-presence networking, antimicrobial resistance and metabolic functional profiling. This study therefore provides an opportunity to investigate the microbiome composition and diversity, and their genomic potentials under controlled anaerobic condition through shotgun metagenomic sequencing, and also to relate their functional activities to generate renewable energy under changing environmental condition and processing parameters.

## Results

### Physicochemical properties of substrate and digesta

**Figure 1:**
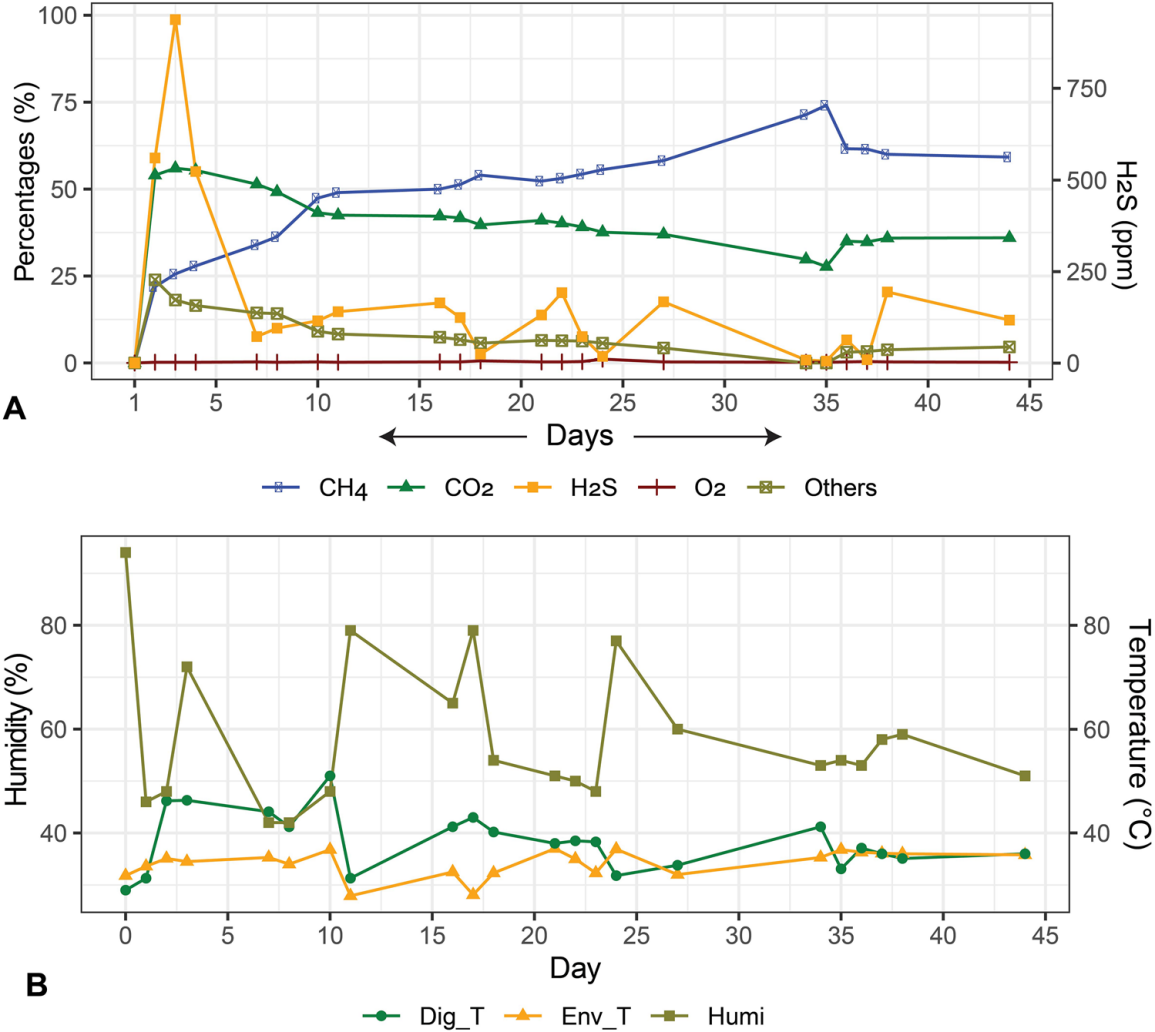
Dynamic changes in the physicochemical parameters of the anaerobic digester (AD) over the study period. The R package, ggplot2 (https://cran.r-project.org/web/packages/ggplot2/index.html) was used to visualize the line graphs.

**Figure 2:**
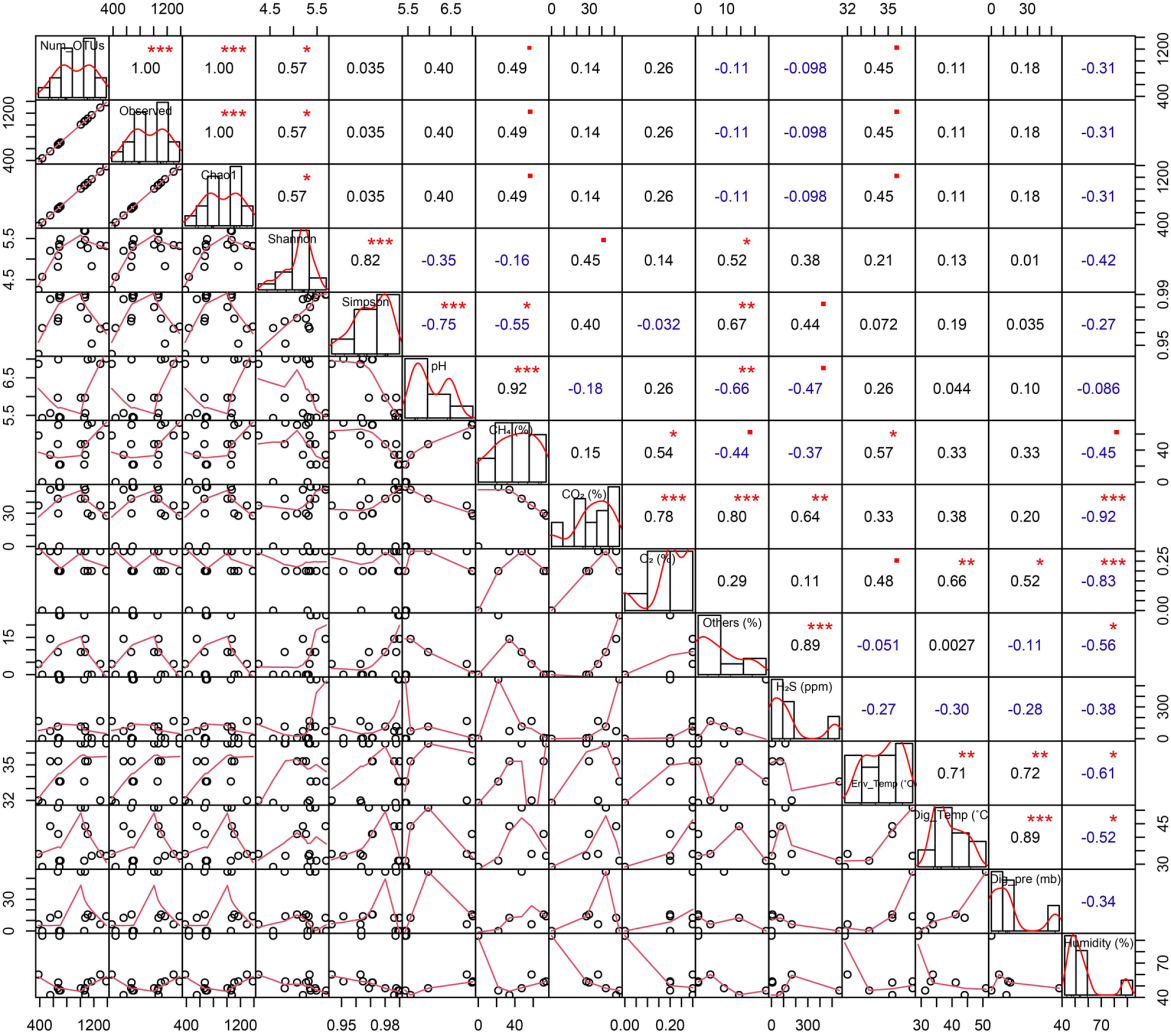
Pairwise Pearson’s correlation between each of the two physicochemical and microbial alpha diversity characteristics. Black and blue indicate positive and negative correlation, respectively. The figures demonstrate the scale of correlation. pH, CH_4_ (%), CO_2_ (%), O_2_ (%), Others (%), H2S (ppm), Env_Temp (°C) (Environmental_Temperature), Dig_Temp (°C) (Digester_Temperature), Dig_pre (mb) (Digester_pressure), Humidity (%) are different physicochemical parameters. Num_OTUs are the numbers of microbial OUTs. Observed, Chao1, Shannon, and Simpson are the α-diversity indices. *Significant level (■ p < 0.1; *p < 0.05; **p < 0.01; ***p < 0.001). The R package, chart. Correlation function of PerformanceAnalytics (https://cran.r-project.org/web/packages/PerformanceAnalytics/index.html) was used to analyze and visualize the plot.

We observed distinct differences in the physicochemical properties of the digester feedstock throughout the experiment which, thus, could have affected the digester performance. The physicochemical properties of the digester feedstock before and after the anaerobic digestion of CD are shown in Fig. 1. The fermentation process of the AD was run for 45 days (Day 0 to Day 44) with a periodic loading of raw CD. The mean loading dose of the raw CD was 90.56 kg/load (highest input volume = 375 kg and lowest input volume = 35 kg) (Table S1). Periodic increments of the raw CD resulted in increased biogas production (Fig. 1A, Table S1). This trend was observed until Day 35, after which biogas production began to decline gradually, although the CD loading was continued. The log phase of methane (CH_4_) production started around the second day of the experiment, and reached its maximum percentage (74.1%) at Day 35 of the digestion process. It should be pointed out that after Day 35, the CH_4_ percentage started to decrease, reaching 59.2% on Day 44 (Table 1). Average concentration (%) CO_2_ was observed 39.52 (minimum = 27.7, maximum = 56) throughout 44 days of the digestion process. Concentration of H_2_S was maximum (938 ppm) at Day 3, and later on the concentration fluctuated based on feeding (Fig. 1A, Table S1). The overall environmental temperature, AD temperature, AD pressure and humidity were 34.75 °C (maximum = 38.8 °C, minimum = 32.0 °C), 34.46 °C (maximum = 51.0 °C, minimum = 0.0 °C), 22.52 mb (maximum = 56.41 mb, minimum = 0.0 mb), and 55.5% (maximum = 94.0%, minimum = 42.0%) (Fig. 1B, Table S2). On Day 35 of the digestion, when maximum methanogenesis was observed, the concentration of organic carbon (OC) and total nitrogen (TN) in the fermentation pulp were 15.48% and 1.22%, respectively, whereas the concentration of OC and TN in the slurry (CD + seed sludge; Day-0) were 34.39% and 1.96%, respectively (Table 2). The overall C/N ratio of the feedstock also gradually decreased with the advent of anaerobic digestion process, and found lowest (12.7:1) at Day 35. Similarly, the amount of non-metallic element (phosphorus and sulfur) and heavy metals (chromium, lead and nickel) content significantly decreased at the Day 35 of the digestion process (Table 2). However, the amount of zinc and copper did not vary significantly throughout the digestion period (Table 2). As shown in Fig. 2, number of operational taxonomic units (OTUs) were significantly positively correlated with AD bacterial α-diversity (Observed, Chao1 and Shannon indices; r were 1.00, 1.00, and 0.57, respectively, p < 0.05). Similarly, we found significant positive correlation between CH_4_ concentration (%) and pH (r = 0.92, p < 0.001), CH_4_ concentration (%) and O_2_ level (%) (r = 0.54, p < 0.05), and CH_4_ concentration (%) and environmental temperature (°C) (r = 0.57, p < 0.05) (Fig. 2). We also found positive correlation between the AD CO_2_ (%) and H_2_S (%), environmental temperature and digester temperature, environmental temperature and digester pressure, digester temperature and digester pressure (r = 0.64, p < 0.01; r = 0.71, p < 0.01; r = 0.72, p < 0.01; r = 0.89, p < 0.01, respectively). Interestingly, we also found a negative correlation between CO_2_ and humidity, O_2_ and humidity, environmental temperature and humidity, and digester temperature and humidity (Fig. 2).

**Table 1:** Metagenome samples and their groupings in the AD according to experiment time and methane (CH_4_) concentration up to Day 35 of digestion (highest methane production level).

Groups	Day	CH_4_ (%)	pH	DNA purity and concentration			Reads after QC	No. of OTUs	SRA accession
				Sample ID	Purity (280/260)	Concentration (ng/µL)			
Group-I	0	0	5.43	R1	1.78	103.7	20,962,184	437	SRR12814733
			5.46	M3	1.82	91.7	21,439,832	690	SRR12814732
Group-II	2	21	5.44	O4	1.88	61	22,222,964	687	SRR12814725
				I5	1.88	77.7	18,879,852	706	SRR12814724
				M6	1.87	73.4	22,207,206	1058	SRR12814723
	7	34	5.56	O7	1.87	46	21,742,834	1073	SRR12814722
				I9	1.88	87.2	22,074,694	555	SRR12814721
Group-III	10	47.4	5.97	O10	1.87	48.6	21,769,978	1069	SRR12814720
				M11	1.86	86.1	20,872,498	1007	SRR12814719
				I12	1.88	100.5	23,074,762	667	SRR12814718
	27	58.2	6.87	O60	1.89	59.4	22,044,170	1295	SRR12814731
				O13	1.89	61.3	18,850,242	380	SRR12814730
Group-IV	34	71.4	6.99	O15	1.81	67.7	21,713,262	1109	SRR12814729
				I14	1.82	65.4	22,361,088	673	SRR12814728
	35	74.1	7.01	O16	1.81	112.3	22,044,170	1168	SRR12814727
				I17	1.81	53.6	21,394,606	1384	SRR12814726

*R1 = Raw cow dung, M3 = mixtures with raw cow dung and slurry from previous biogas plant as seed, O = Outlet samples, I = Input position samples, M = Middle position samples.

**Table 2:** Physicochemical properties of raw cow dung, slurry and active sludges.

Parameters	Cow dung (CD; Day-0)	Slurry (CD + seed sludge; Day-0)	Active sludge (AS; Day-35, highest CH_4_ concentration)
Moisture (%)	23.32	86.16	90.03
Organic carbon (%)	34.39	36.83	15.48
Total nitrogen (%)	1.82	1.96	1.22
C: N	18.9:1	18.8:1	12.7:1
Phosphorus (%)	0.249	0.461	0.070
Sulphur (%)	0.522	0.579	0.038
Zinc (mg/kg)	16.24	17.56	15.58
Copper (mg/kg)	2.88	3.59	2.13
Chromium (mg/kg)	11.97	11.33	0.23
Cadmium (mg/kg)	BDL	BDL	0.04
Lead (mg/kg)	3.44	3.26	1.06
Nickel (mg/kg)	3.28	6.70	0.34

CD: raw semi-solid cow dung which was mixed with water; Slurry: mixture of CD and seed sludge from previous biogas plant; AS: slurry from the AD when the gas production rate was the highest (i.e. 74.1% CH_4_ concentration); and BDL: below detection limit.

### Microbiome composition and diversity in the AD

**Figure 3:**
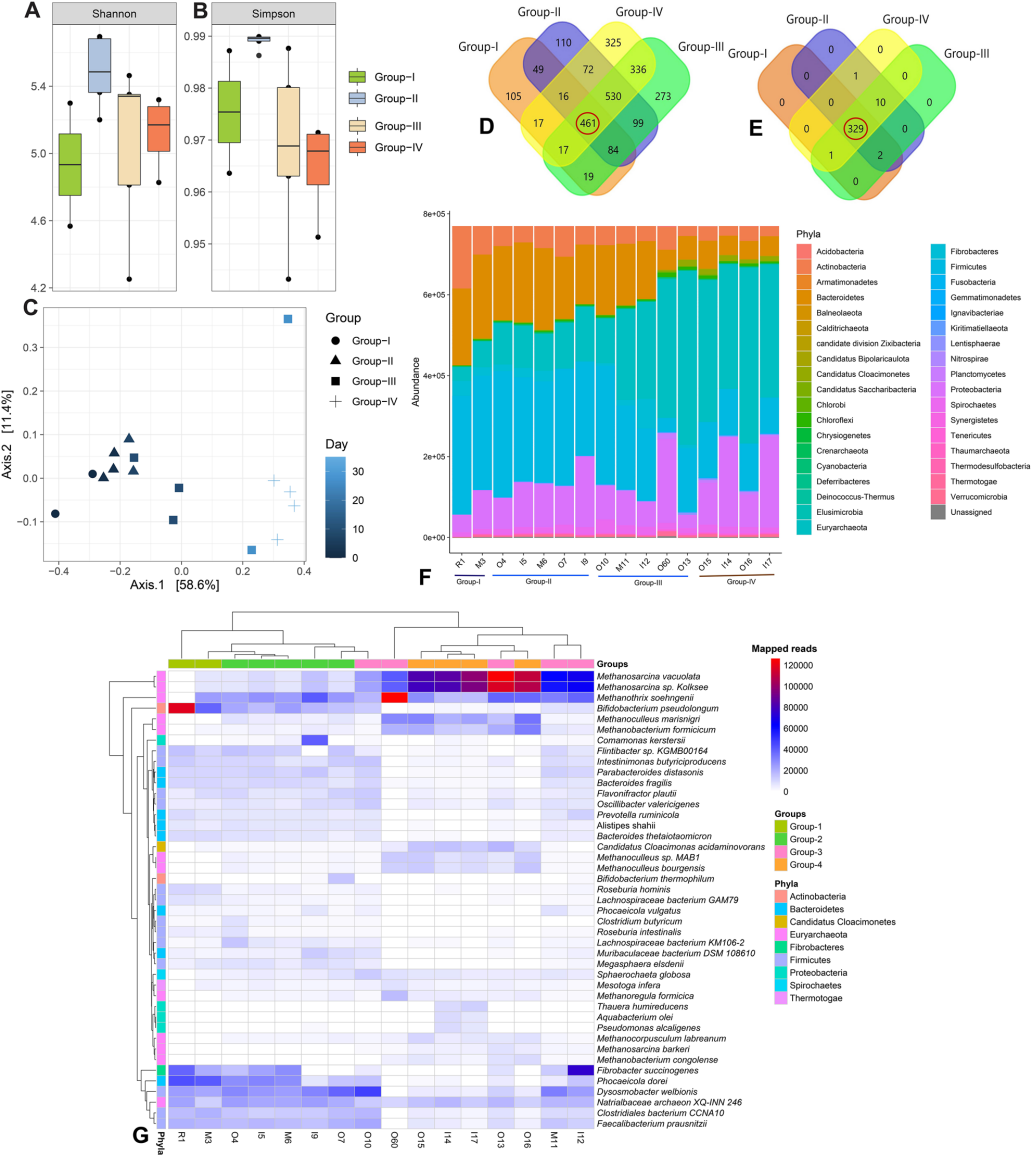
Microbiome diversity, composition and distribution in four metagenomic groups of the anaerobic digestate samples. (**A-B**) Box plots showing significant differences in observed species richness in AD associated microbiome. Alpha diversity, as measured by PathoScope (PS) analysis using the Shannon and Simpson diversity indices, revealed distinct microbiome diversity across four metagenome samples (p = 0.03541, Kruskal–Wallis test). (**C**) The experimental groups were clearly separated by principal coordinate analysis (PCoA), which was measured using non-metric multidimensional scaling (NMDS) ordination plots. The different shapes represent the assigned populations in four metagenomes. As the day progresses, the group color becomes lighter. Values in parentheses represent the fraction of the total variance explained by each PCoA axis. (**D**) Venn diagram showing unique and shared bacterial strains, (**E**) Venn diagram showing unique and shared archaeal strains. (**F**) Stacked bar plots showing the relative abundance and distribution of the total microbial community (phyla level), The archaeal phylum, *Euryacaeota* showed an increasing trend from initial stage to the final stage (Group-IV) of digestion. (**G**) Dominant microbial community (≥ 1% reads at least one sample) at species level were visualized in heatmap. Forty-three dominant species were found in this study. Red, blue, and white colors represent different mapped reads to specific taxa from higher to lower respectively. FunRich (http://funrich.org/) tool was used to draw Venn diagram. Data were processed through Phyloseq (https://www.bioconductor.org/packages/release/bioc/html/phyloseq.html) R package, visualized by using ggplot2 (https://cran.r-project.org/web/packages/ggplot2/index.html). Heatmap was generated by using Pheatmap (https://cran.r-project.org/web/packages/pheatmap/pheatmap.pdf) R package.

The samples (n = 16) were categorized into four groups (Group-I, Group-II, Group-III and Group-IV) based on the level of energy production (CH_4_%) (Table 1). Group-I (n = 2) belonged to initial time of energy production, Group-II (n = 5) and Group-III (n = 5) had 21–34% and 47–58% of CH_4_ concentration, respectively. The samples having the highest 71–74% of CH_4_ was categorized into Group-IV (n = 4). The WMS of 16 sample libraries resulted in 380.04 million reads passing 343.26 million reads quality filters, which corresponded to 90.32% total reads (individual reads per sample are shown in Data S1). The major microbial domain in all samples was Bacteria with an abundance of 81.80%, followed by Archaea (15.43%), and Viruses (2.77%) (Data S1). From these WMS reads, 380–1384 OTUs (average = 850 ± 22) were identified representing the distribution of microbial taxa cross four groups of the AD. Remarkably, the Group-IV metagenome always had higher number of OTUs compared to other groups (Table 1). The alpha diversity (i.e., within-sample diversity) of the AD microbiomes was computed using the Shannon and Simpson estimated indices (i.e., a diversity index accounting both evenness and richness) at the strain level. In this study, both Shannon and Simpson indices estimated diversity significantly varied across the four sample groups (p = 0.03541, Kruskal–Wallis test). The pair-wise comparison of the within sample diversity revealed that the microbiomes of the Group-II significantly differed with those of Group-III and Group-IV (p = 0.048, Wilcoxon rank sum test for each) compared to Group-I (p = 0.91, Wilcoxon rank sum test) (Fig. 3A, B). The rarefaction analysis of the observed species showed a plateau after, on average, 21.45 million reads (Fig. S2, Data S1)-indicating that the coverage depth for most samples was sufficient to capture the entire microbial diversity. We also observed significant differences in the microbial community structure among the four metagenome groups (i.e., beta diversity analysis). Principal coordinate analysis (PCoA) at the strain level (Fig. 3C), showed a distinct separation of samples by the experimental groups. Besides, we found significant (p = 0.032, Kruskal Wallis test) differences in the abundance of ARGs and metabolic functional genes/pathways (DataS2) which could strongly modulate the level of energy production through microbiome dysbiosis in the AD.

In this study, on an average 0.43% WMS reads (assigned for r RNA genes) mapped to 28, 110 and 552 bacterial phyla, orders and genera respectively, and relative abundance of the microbiome differed significantly (p = 0.034, Kruskal–Wallis test) across the metagenome groups (Data S1). We observed significant shifts/dysbiosis in the microbiome composition at strain level. The PS analysis detected 2,513 bacterial strains across the four metagenomes, of which 768, 1421, 1819 and 1774 strains were found in Group-I, Group-II, Group-III and Group-IV metagenomes, respectively. Only, 18.34% bacterial strains were found to be shared across the four energy producing metagenomes (Fig. 3D, Data S1). The archaeal fraction of the AD microbiomes was represented by 5, 17, 61 and 343 archaeal phyla, orders, genera and strains, respectively, and the relative abundance of these microbial taxa also varied significantly among the four metagenome groups (Fig. 3E). Remarkably, 95.90% (329/343) of the detected archaeal strains shared across these metagenomes (Fig. 3E, Data S1). In addition, 472, 536, 535 and 536 strains of bacterial viruses (bacteriophages) were identified in Group-I, Group-II, Group-III and Group = IV metagenomes, respectively (Data S1).

### Microbial community dynamically changed over time in the AD

Significant changes in the abundances of core microbial groups were observed under anaerobic condition of the AD. At phylum level, the AD metagenome was dominated by *Firmicutes*, *Bacteroidetes*, *Proteobacteria*, *Actinobacteria*, *Spirochaetes* and *Fibrobacteres* comprising > 93.0% of the total bacterial abundances. Among these phyla, *Firmicutes* was the most abundant phylum with a relative abundance of 41.94%, 37.99%, 40.40% and 38.96% in Group-1, Group-II, Group-III and Group-IV, respectively. The relative abundance of *Bacteroidetes* (from 37.87% in Group-I to 22.40% in Group-IV) and *Actinobacteria* (from 3.94% in Group-I to 3.30% in Group-IV) gradually decreased with the advance of AD digestion time. Conversely, relative abundance of *Proteobacteria* (from 8.08% in Group-I to 18.92% in Group-IV) and *Spirochaetes* (from 1.28% in Group-I to 3.70% in Group-IV) gradually increased with the increase of anaerobic digestion time in AD. The rest of phyla also differed significantly across these four groups keeping comparatively higher relative abundances during highest CH_4_ producing stage (Group-IV) of the AD (Fig. 3F). The relative abundances of archaeal phylum *Euryarchaeota*, steadily increased with the increasing demand of energy (lowest relative abundance in Group-I and highest relative abundance in Group-IV) (Fig. 3F). Similarly, *Clostridiales* and *Bacteroidales* were identified as the top abundant order in Group-1, Group-II, Group-III and Group-IV with a relative abundance of 32.37%, 27.81%, 29.22% and 27.87%, and 32.49%, 27.42%, 27.53% and 14.94%, respectively (Data S1).

The structure and relative abundances of the bacteria at the genus level also showed significant differences (p = 0.031, Kruskal–Wallis test) across the study groups. In Group-I, Group-II and Group-III metagenomes, *Bacteroides* was the most abundant bacteria with a relative abundance of 18.10%, 14.90% and 15.16%, respectively, but remained lower (8.31%) in Group-IV samples. *Clostridium* was found as the second most predominant bacterial genus, and the relative abundance of this bacterium was 11.92%, 11.13%, 11.73% and 12.15% in Group-1, Group-II, Group-III and Group-IV, respectively. The relative abundance of *Ruminococcus*, *Eubacterium*, *Parabacteroides*, *Fibrobacter*, *Paludibacter*, *Porphyromonas* and *Bifidobacterium* gradually decreased with the increase of energy (CH_4_) production rate, and remained lowest in Group-IV. Conversely, *Candidatus*, *Bacillus*, *Treponema* and *Geobacter* showed an increasing trend in their relative abundances gradually with the advance of digestion time and remained lowest in relative abundances in Group-IV. The rest of the bacterial genera had lower relative abundances in four metagenomes of the AD (Fig. S3, Data S1). In our present study, *Methanosarcina* was the most abundant archaeal genus, and the relative abundance of this genus remained two-fold higher in Group-III (35.84%) and Group-IV (36.53%) compared to Group-I (17.52%) and Group-II (18.32%). Notably, the relative abundance of *Methanoculleus* was found higher in Group-II (11.59%) and Group-IV (13.80%) and lowest in Group-I (3.46%). Likewise, *Methanobrevibacter* was predominantly abundant at the initial phage of digestion (highest in Group-I; 19.35%) and remained lowest in abundance in the top CH_4_ producing metagenome (Group-IV; 5.01%). Besides these genera, *Methanothermobacter* (5.30%), *Methanosaeta* (5.16%), *Methanococcus* (4.74%), *Thermococcus* (2.96%), *Methanocaldococcus* (2.53%), *Pyrococcus* (2.35%), *Methanosphaera* (2.32%) *Methanococcoides* (2.10%) and *Archaeoglobus* (2.01%) were the predominantly abundant archaeal genera in Group-I samples and their relative abundances gradually decreased with the increase of energy production (Fig. S4, Data S1). On the other hand, *Methanoregula* (6.43%), *Methanosphaerula* (2.99%), *Methanoplanus* (2.37%) and *Methanohalophilus* (1.39%) were the most abundant archaeal genera in Group-IV metagenome. The rest of the genera remained much lower (< 1.0%) in relative abundances but varied significantly across the four metagenomes (Fig. S4, Data S1).

The strain-level composition, diversity and relative abundances of the microbiomes across four metagenomes revealed significant variations (p = 0.011, Kruskal–Wallis test) (Data S1). In this study, 2,513 bacterial and 343 archaeal strains were detected, of which 18.35% (461/2513) bacterial and 95.92% archaeal strains shared across the study metagenomes (Fig. 3, Data S1). Most of the bacterial strains detected were represented by the phylum *Firmicutes* followed by *Bacteroidetes*, *Gammaproteobacteria* and *Betaproteobacteria* (Fig. S5, Data S1). Of the detected strains, methanogenic archaeal strains were more prevalent (higher relative abundances) compared to bacterial strains, and this stain-level microbiome profiling was more evident in highest energy producing metagenome group (Group-IV). The most prevalent energy producing archaeal strains in Group-IV were *Methanosarcina vacuolata* Z-761 (17.31%), *Methanosarcina* sp. Kolksee (16.63%), *Methanoculleus marisnigri* JR1 (5.0%), *Methanothrix soehngenii* GP6 (4.61%), *Methanobacterium formicicum* DSM 1535 (3.60%), *Methanoculleus* sp. MAB1 (2.07%) and *Methanoculleus bourgensis* DSM 3045 (2.07%), and rest of strains had lower (< 2.0%) relative abundances (GData S1). Moreover, the relative abundances of these strains gradually increased with the increase of energy production (lowest relative abundance in Group-I and highest relative abundance in Group-IV) (Data S1). Conversely, the relative abundances of most of the bacterial strains identified gradually decreased with the advance of digestion time (increase of energy production), and mostly remained higher in relative abundances in Group-I (Data S1). Of the top abundant bacterial strains, *Bifidobacterium pseudolongum* subsp. globosum DSM 20,092 (12.0%), *Phocaeicola dorei* DSM 17,855 (6.61%), *Fibrobacter succinogenes* subsp. succinogenes S85 (4.57%), *Faecalibacterium prausnitzii* M21/2 (2.89%), *Clostridiales bacterium* CCNA10 (2.78%), and *Flintibacter* sp. KGMB00164 (2.07%) were found in Group-I, and their abundances gradually decreased with the increase of level of CH_4_ production. In addition, *Dysosmobacter welbionis* J115 (5.48%) remained more prevalent in Group-II (Data S1). The rest of the bacterial strains were less abundant (< 2.0%) across the four metagenomes (Data S1). The viral fraction of the microbiomes mostly dominated by different strains of bacteriophages such as *Gordonia* phage Secretariat (16.12%), *Streptomyces* phage Bing (5.33%) and *Arthrobacter* phage Gordon (5.05%) in Group-I, *Megavirus chiliensis* (1.81%), *Acanthamoeba polyphaga* moumouvirus (1.60%) and *Orpheovirus* IHUMI-LCC2 (1.50%) in Group-II, *Stenotrophomonas* phage Mendera (4.88%), *Choristoneura fumiferana* granulovirus (3.0%) and *Gordonia* phage Secretariat (2.55%) in Group-III and *Stenotrophomonas* phage Mendera (2.58%), *Choristoneura fumiferana* granulovirus (2.37%) and *Bacillus* phage Mater (1.47%) in Group-IV (Data S1).

### Dominant microbial consortia in the AD

**Figure 4:**
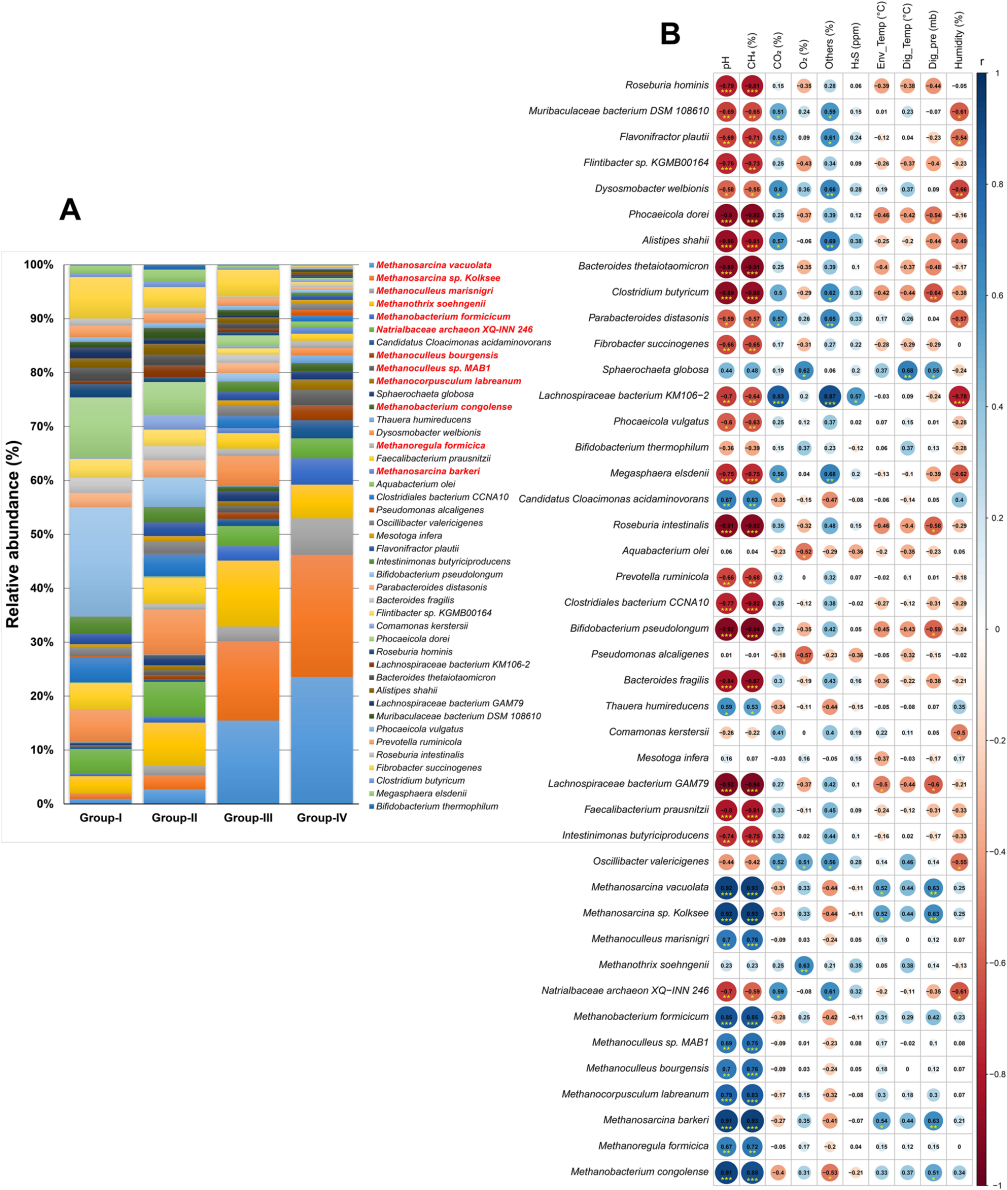
Microbial abundance and the correlation between different physicochemical parameters and microbial relative abundance. (**A**) The species-level taxonomic abundance of microbiome. Stacked bar plots showing the relative abundance and distribution of the dominant abundant species (43 taxa), with ranks ordered from bottom to top by their increasing proportion among the four metagenomics groups. Each stacked bar plot represents the abundance of each strain in each sample of the corresponding category. The relative abundances of archaeal species (red color legends) steadily improved as energy demand increased (lowest relative abundance in Group-I and highest relative abundance in Group-IV). In contrast, the relative abundances of most of the known bacterial species gradually decreased with the passage of time (increased energy production), and mostly remained higher in Group-I and lower in Group-IV. (**B**) Spearman’s correlation analysis between different physicochemical parameters [pH, CH_4_ (%), CO_2_ (%), O_2_ (%), Others (%), H2S (ppm), Env_Temp (°C) (Environmental_Temperature), Dig_Temp (°C) (Digester_Temperature), Dig_pre (mb) (Digester_pressure), Humidity (%)] and dominant microbial relative abundance at species level. The numbers display the Spearman’s correlation coefficient (r). Blue and red colors indicate positive and negative correlation, respectively. The color density, circle size, and numbers reflect the scale of correlation. *Significant level (*p < 0.05; **p < 0.01; ***p < 0.001). The R packages, Hmisc (https://cran.r-project.org/web/packages/Hmisc/index.html) and corrplot (https://cran.r-project.org/web/packages/corrplot/vignettes/corrplot-intro.html) were used respectively to analyze and visualize the data.

In the present study, we detected 43 dominant species including 31 bacterial and 12 archaeal species belonged of 9 phyla (8 bacterial and 1 archaeal) (Fig. 3G, Data S1). In the digestate of AD, *Firmicutes*, *Bacteroidetes*, *Fibrobacteres*, *Spirochaetes*, *Actinobacteria*, *Candidatus Cloacimonetes*, *Proteobacteria*, and *Thermotogae* were the dominant bacterial phyla while *Euryarchaeota* was the only archaeal phylum dominating in the AD. Methanogenic archaeal species were dominant in Group-III and Group-VI (*M. vacuolata*, *Methanosarcina* sp. Kolksee, *M. marisnigri*, *M. soehngenii*, *M. formicicum* and *Natrialbaceae archaeon* XQ-INN 246) (Fig. 4A). Most of the dominant bacterial species had negative correlation with digester pH, CH_4_, humidity and pressure. Of the detected dominant species, most of the dominating methanogenic archaeal species such as *M. vacuolata*, *Methanosarcina* sp. Kolksee, *M. marisnigri*, *M. soehngenii*, *M. formicicum*, *Methanoculleus* sp. MAB1, *M. bourgensis*, *M. labreanum*, *M. barkeri*, *M. formicica* and *M. congolense* showed strongest positive correlation with pH, CH_4_ (%), digester pressure (%) and temperature (°C) (Spearman correlation; r > 0.6, p < 0.01) (Fig. 4B). On the other hand, bacterial species including *Roseburia hominis*, *Flintibacter* sp., *P. dorei*, *Alistipes shahii*, *B. thetaiotaomicron*, *C. butyricum*, *Megasphaera elsdenii*, *R. intestinalis*, *C. bacterium* CCNA10, *B. pseudolongum*, *B. fragilis*, *L bacterium* GAM79, *F. prausnitzii* and *I. butyriciproducens*, and archaea species *N. archaeon* XQ-INN 246 had significant negative correlation with digester pH, CH_4_ concentration (%) and humidity (%) (Spearman correlation; r > 0.6, p < 0.01) (Fig. 4B).

### Identification of potential indicator species and their co-occurrence

**Figure 5:**
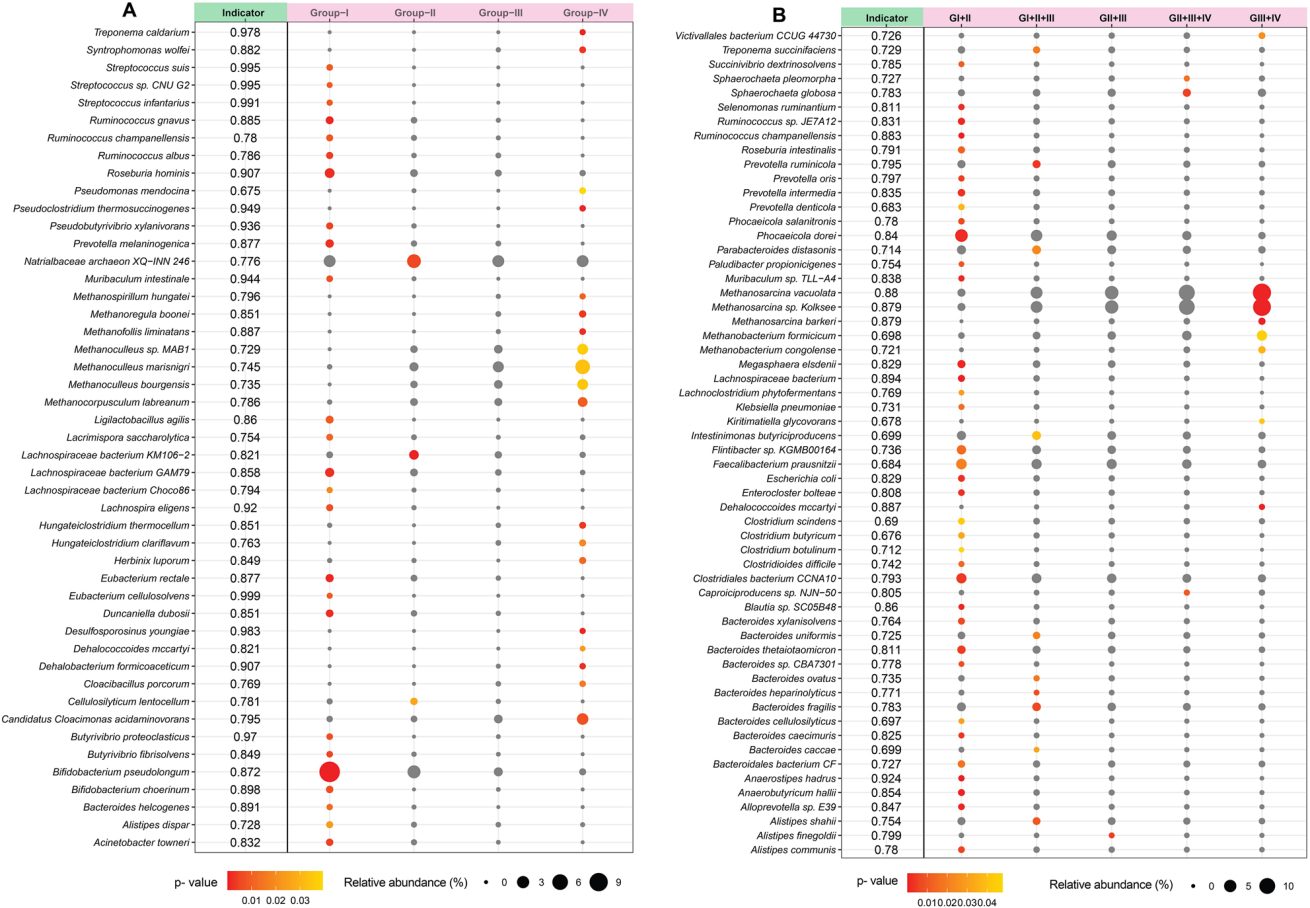
Indicator species analysis of AD microbiome within four metagenomics groups. (**A**) Individual group effects of the indicator species associated with energy production, (**B**) combined group effects of the indicator species associated with energy production. Indicator values (IndVal) plotted next to the name of taxa range from zero to one, with larger values denoting greater specificity. Indicator values (IndVal) are shown next to the taxonomic information for the indicator taxa as indicated by Indicator. Size of symbol ((circle size) is proportional to the mean relative abundance in that group of AD and p-values (circle color) were plotted. Circles colored in grey indicate insignificant taxa. Higher indicator values (IVs) suggested better performances in the microbial signature of the assigned taxa. The R package, Indicspecies (https://cran.r-project.org/web/packages/indicspecies/index.html) was used to analyze the data, and ggplot2 (https://cran.r-project.org/web/packages/ggplot2/index.html) was used to draw these bubble groups.

To identify microbial taxa (bacteria and archaea) that could discriminate across the four metagenome groups of the AD in terms of energy production (% CH_4_), the indicator species analysis (ISA) was performed both in individual group and combination basis, as shown in (Fig. 5). Indicator species were those which were significantly more abundant and present in all samples belonging to one group, and also absent or low abundance in the other group (Fig. 5, Data S1). The core taxa were selected based on their relative frequency (≥ 75% occurrence in each of the four groups) (Data S1). Although, 26, 3 and 19 indicator species were found in Group-I, Group-II and Group-IV, respectively, and no indicator species were identified in Group-III (Fig. 5A, Data S1). Higher indicator values (IVs) suggested better performances in the microbial signature of the assigned taxa. *Desulfosporosinus youngiae*, *Treponema caldarium*, *Pseudoclostridium thermosuccinogenes*, *Dehalobacterium formicoaceticum*, *Methanofollis liminatans*, *Methanoregula boonei*, *Syntrophomonas wolfei*, *Hungateiclostridium clariflavum*, *Candidatus Cloacimonas acidaminovorans* and *Methanocorpusculum labreanum* were highly specific for energy production in Group-IV (highest CH_4_ production rate; 74.1%), with IVs of 0.983, 0.978, 0.949, 0.907, 0.887, 0.885, 0.882, 0.851, 0.795 and 0.786, respectively (Fig. 5A; Data S1). Considering the combined group effects of the indicator species associated with energy production, our analysis revealed that *Methanosarcina vacuolate*, *Dehalococcoides mccartyi*, *Methanosarcina* sp. Kolksee and *Methanosarcina barkeri* in Group-III + Group-IV (top CH_4_ producing groups) having IVs of 0.88, 0.887, 0.879 and 0.879, respectively were highly specific for energy production (Fig. 5B, Data S1). All of the indicator phylotypes displayed reduced abundance in the initial stage of biogas production (Group-I and Group-II, lower CH_4_ production rate) compared to their increased relative abundance up to Day 35 of the experiment (in Group-III and Group-IV) (Data S1).

**Figure 6:**
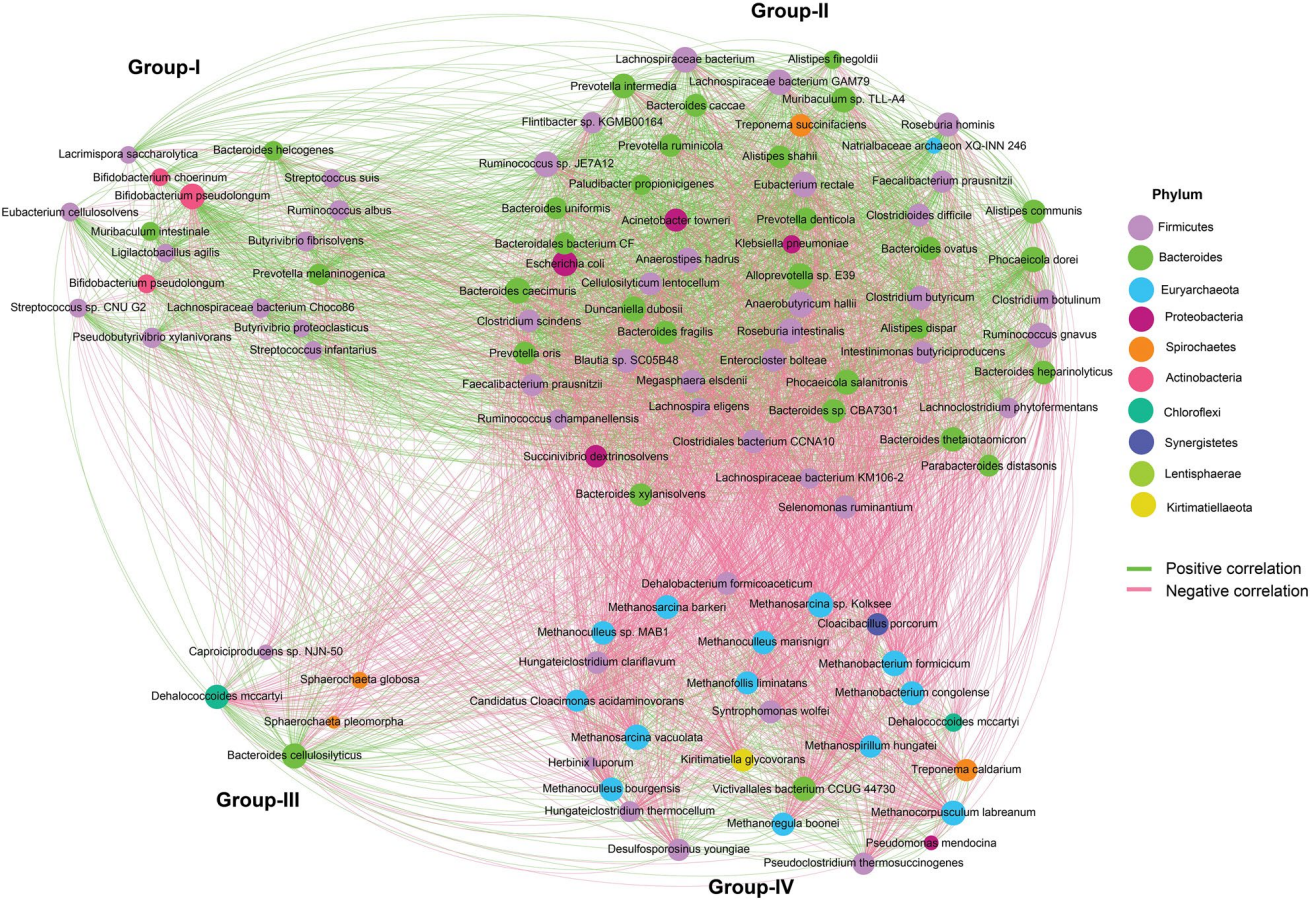
Microbiome co-occurrence in the AD within the four metagenomic groups. Correlation networks of significantly altered species based on the indicator species analysis. Spearman’s absolute correlation coefficients ≥ 0.5 and p-values < 0.05 were retained. The node size is proportional to the mean abundance of the species. Nodes are colored by taxonomy with labelled genera names. The positive correlation is represented by the green line, while the negative correlation is represented by the red line. The nodes were grouped according to their higher abundance in each group (It means that a species can be found in all or at least one group. But they were kept in such a group where it was highly abundant). The microbiomes of the AD showed positive associations between Groups I and II, while Groups III and IV showed negative correlations in terms of energy production with the microbial taxa of the other two groups. The R packages, Hmisc (https://cran.r-project.org/web/packages/Hmisc/index.html) package was used to analyze correlation and Gephi (https://gephi.org/) software was utilized to visualize the network plot.

We then visualized networks within each metagenome group of the AD for both positive and negative co-occurrence relationships (Fig. 6, Data S1). The correlation networks analysis was performed based on the significantly altered species (n = 106) in different groups as revealed by indispecies analysis. This network analysis explored significant association (p = 0.021, Kruskal–Wallis test) in the co-occurrence patterns of the energy producing microbial taxa (species and/or strains) based on their relative abundances in four metagenome groups. In the correlation network of four metagenomes; Group-I to Group-IV), *Firmicutes* and *Bacteroidetes* exhibited strongest relation. The resultant network consists of 106 nodes (17 in Group-I, 58 in Group-II, 5 in Group-III and 26 in Group-IV) which were clearly separated into four modules/clusters (Fig. 6). Taxa in the same group may co-occur under the same AD conditions (temperature, O_2_ and H_2_S percentage, pressure and humidity). Across different metagenome groups of AD, *Firmicutes*, *Bacteroidetes*, *Actinobacteria* and *Proteobacteria* were the top abundant phyla in Group-I and Group-II with a cutoff of 1.0 while *Bacteroidetes* and *Chlorhexi* in Group-III, and *Euryarcheota* and *Firmicutes* in Group-IV were designated as the top abundant phyla with a cutoff of 1.0 (Fig. 6). However, when moving down to the species-level in microbiome co-occurrence in the AD, keystone taxa were much more consistent between networks with different correlation cutoffs. These results reveal that applying the same conditions in the AD for energy production, network elements must happen under careful consideration of the parameters used to delineate co-occurrence relationships. The positive correlations between Group-I and Group-II were observed among the microbiomes of the AD while Group-III and Group-IV showed negative correlations in terms of energy production with the microbial taxa of other two groups (Fig. 6). These findings therefore suggest that different strains of *Euryarcheota* and *Firmicutes* phyla were negatively correlated but associated with highest level of energy production (highest % of CH_4_; Group-IV).

### Genomic functional potentials of the anaerobic microbiomes

**Figure 7:**
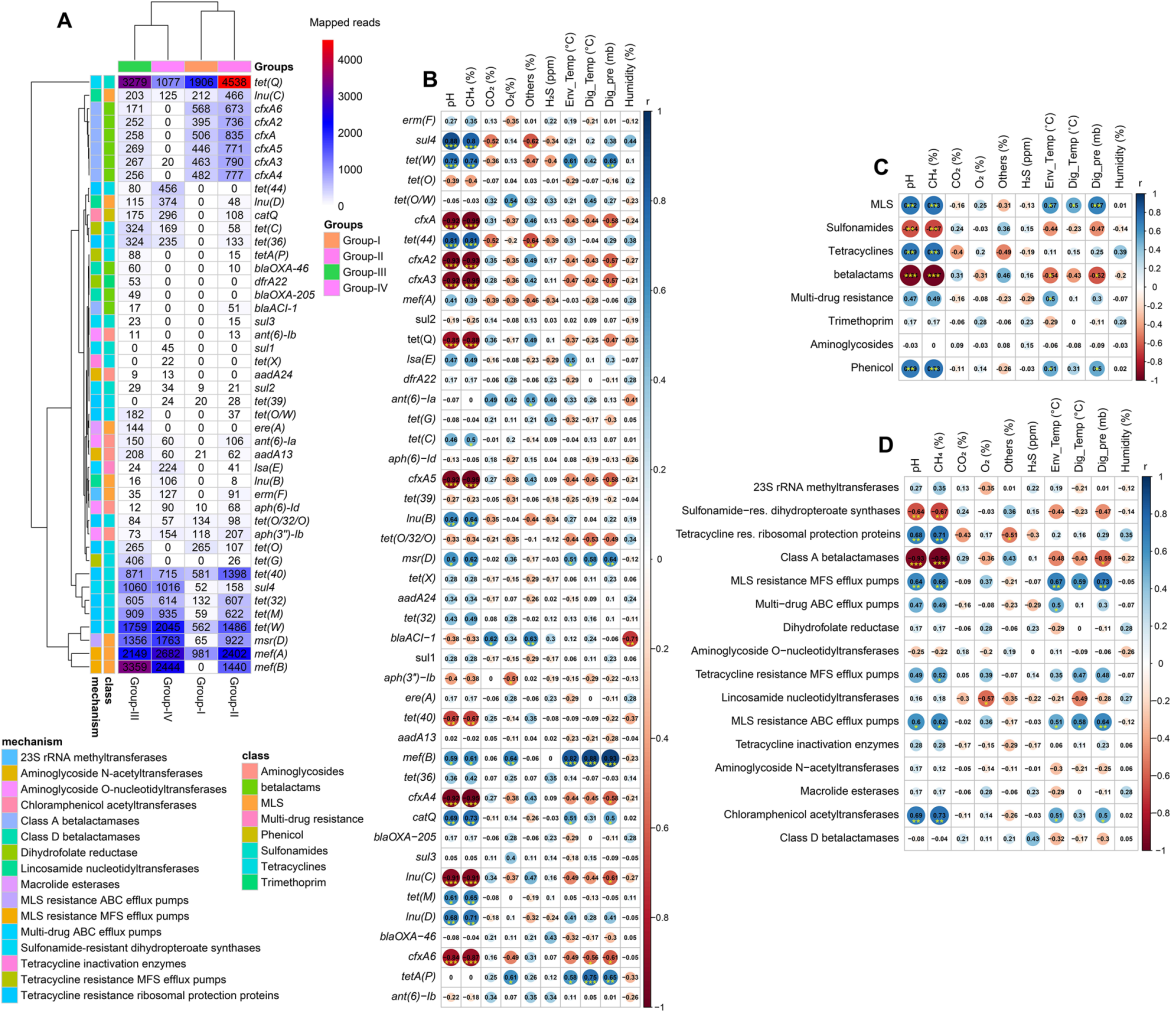
Antibiotics resistance genes (ARGs) detected in anaerobic digestion driving microbiome and their Spearman’s correlation with physicochemical parameters and dominant microbial community. (**A**) The heatmap illustrates the distribution of 45 ARGs belonged to 8 antibiotic classes and 16 types of mechanisms found across the four metagenomes. The red, blue, white color determine the number of mapped reads and the number also illustrate the same things. (**B**) Spearman’s correlation between physicochemical parameters and ARGs represents the plot. (**C**) The correlation analysis between physicochemical parameters and ARGs classes. (**D**) The correlation analysis between physicochemical parameters and ARGs mechanisms. The numbers display the Spearman’s correlation coefficient (r). Blue and red indicate positive and negative correlation, respectively. The color density, circle size, and numbers reflect the scale of correlation. *Significant level (*p < 0.05; **p < 0.01; ***p < 0.001).

In this study, there was a broad variation in the diversity and composition of the antimicrobial resistance genes (ARGs) (Fig. 7, Data S2). The results of the present study revealed significant correlation (p = 0.0411, Kruskal–Wallis test) between the relative abundances of the detected ARGs and the relative abundance of the associated bacteria found in four metagenomes (Data S2). ResFinder identified 45 ARGs belonged to eight antibiotic classes distributed in 2,513 bacterial strains (Data S2). The Group-III microbiomes harbored the highest number of ARGs (42), followed by Group-II (38), Group-IV (29) and Group-I (22) microbes (Fig. 7, Data S2). The tetracyclines (doxycycline and tetracycline) resistant gene, *tet*Q had the highest relative abundance (23.81%) in Group-I associated bacteria followed by Group-II (22.85%), Group-III (16.49%) and Group-IV (6.73%)–microbes. Macrolides (erythromycin and streptogramin B) resistant genes such as *mef*A (16.80%), *mef*B (15.32%) and *msr*D (11.10%) had higher relative abundances in highest CH_4_ producing metagenome compared to other metagenome groups (Fig. 7A). The broad-spectrum beta-lactams resistant gene, *cfx*A2-6 was found as the common ARG among the microbiomes of four metagenomes, displaying the highest relative abundance (35.58%) in inoculum (Group-I) microbiota followed by Group-II (23.09%), Group-III (8.02%) and Group-IV (0.14%) microbiomes. The rest of the ARGs also varied in their expression levels across the four metagenomes, being more prevalent in the Group-III microbiomes (Fig. 7A, Data S2).

**Figure 8:**
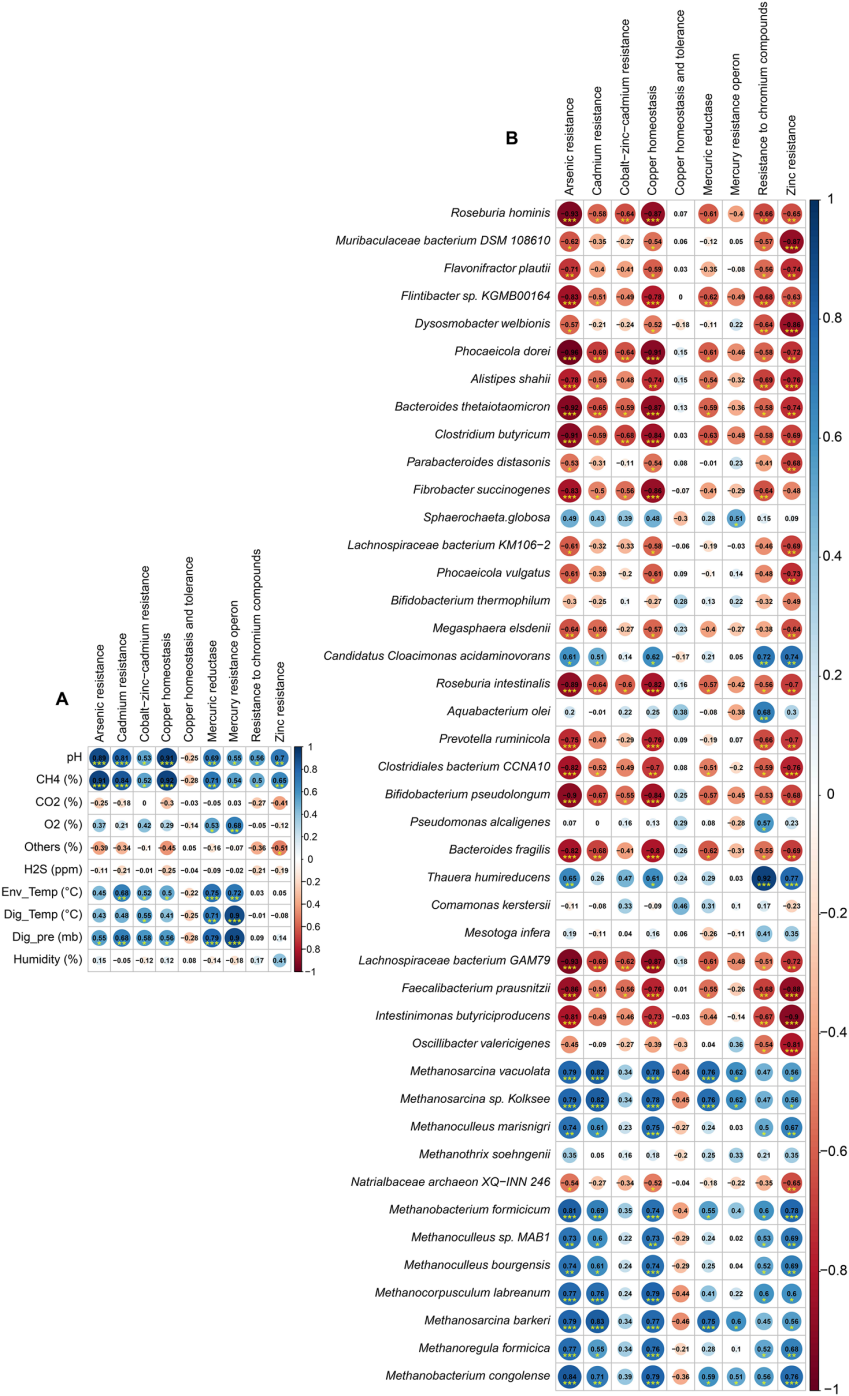
Pairwise Spearman’s correlation with metal resistances (from MG-RAST Seed subsystem) with phytochemical parameters and dominant microbial community. (**A**) Correlation with phytochemical parameters and (**B**) correlation with dominant microbial community. The numbers display the Spearman’s correlation coefficient (r). Blue and red indicate positive and negative correlation, respectively. The color density, circle size, and numbers reflect the scale of correlation. *Significant level (*p < 0.05; **p < 0.01; ***p < 0.001).

Most of the dominant archaeal species had stronger positive correlation with *sul*4, *tet*(W), *tet*(44), *tet*(C), *Inu*(B), *tet*(32), *cat*Q, *tet*M) and *Inu*(D) (Spearman correlation; r > 0.5, p < 0.01), and were negatively correlated with *tet*(Q), *tet*(40), *Inu*(C), *cfx*A, *cfx*A2, *cfx*A3, *cfx*A4, *cfx*A5 and *cfx*A6 (Spearman correlation; r > 0.6, p < 0.01) (Fig. 7B, Data S2, Fig. S6). In contrast, the dominating bacterial species of the AD had significant positive correlation with *cfx*A, *cfx*A2, *cfx*A3, *cfx*A4, *cfx*A5, *cfx*A6, *tet*(Q), *blaACI*-1, *tet*(40) and *Inu*(C) (Spearman correlation; r > 0.6, p < 0.01) (Fig. 7B, Data S2, Fig. S6). Analyzing the correlation between physicochemical parameters and ARGs classes, we found that the digester pH, CH_4_ concentration, pressure and temperature were positively correlated with macrolide-lincosamide-streptogramin (MLS), tetracyclines and phenicol while most of the physicochemical parameters were negatively correlated with sulfonamides class of antibiotics (Spearman correlation; r > 0.6, p < 0.01) (Fig. 7C, Data S2, Fig. S7A). Likewise, the digester pH, CH_4_ concentration, pressure and temperature showed strongest positive correlation with MLS resistance MFS and ABC efflux pumps associated mechanisms (Spearman correlation; r > 0.5, p < 0.01) followed by chloramphenicol acetyltransferases and tetracycline resistance ribosomal protection proteins related functions (Fig. 7D, Data S2). Conversely, Class A betalactamases and sulfonamide resistance dihydropteroate synthases were the negatively correlated mechanisms with digester physicochemical parameters like pH and CH_4_ (Fig. 7D, Data S2). The mechanisms of the antimicrobial resistance also varied between the dominant bacterial and archaeal species (Fig. S7B). For instance, most of the bacterial species showed highest positive correlation Class A betalactamases (Spearman correlation; r > 0.5, p < 0.01) while the enriched archaeal species showed negative correlation with this functional mechanism of antibiotics. The methanogenic archaeal species predominantly identified however had significant positive correlation with tetracycline resistance ribosomal protection proteins related functions, 23S rRNA methyltransferases and MLS resistance MFS efflux pumps (Fig. S7B, Data S2). In addition to these ARGs, the highest CH_4_ producing microbiomes were enriched with the higher relative abundance of genes coding for cobalt-zinc-cadmium resistance (18.85%), resistance to chromium compounds (12.17%), arsenic (6.29%), zinc (4.96%) and cadmium (3.26%) resistance compared to the microbes of other three metagenomes (Fig. S8, Data S2). By comparing the possible mechanisms of the detected ARGs, we found that antibiotic efflux pumps associated resistance had the highest level of expression in the anaerobic microbiomes of the AD followed by antibiotic inactivation, enzymatic inactivation and modification, antibiotic target protection/alteration, and folate pathway antagonist-attributed resistance mechanisms (Fig. S8, Data S2). Notably, the physicochemical properties of the AD including pH, CH_4_ concentration (%), pressure, temperature and environmental temperature were found to be positively correlated with metal such as arsenic, copper, cadmium, mercury, chromium compounds and zinc resistance pathways (Spearman correlation; r > 0.5, p < 0.01). (Fig. 8A, Data S2). Remarkably, most of the methane producing dominant archaeal species showed significant positive correlation with these metal resistance pathways (Spearman correlation; r > 0.7, p < 0.01). (Fig. 8B, Data S2) while the predominant bacterial species to be associated with methane production showed stronger negative correlation with most of the metal resistance pathways (Spearman correlation; r > 0.7, p < 0.01). (Fig. 8B, Data S2).

**Figure 9:**
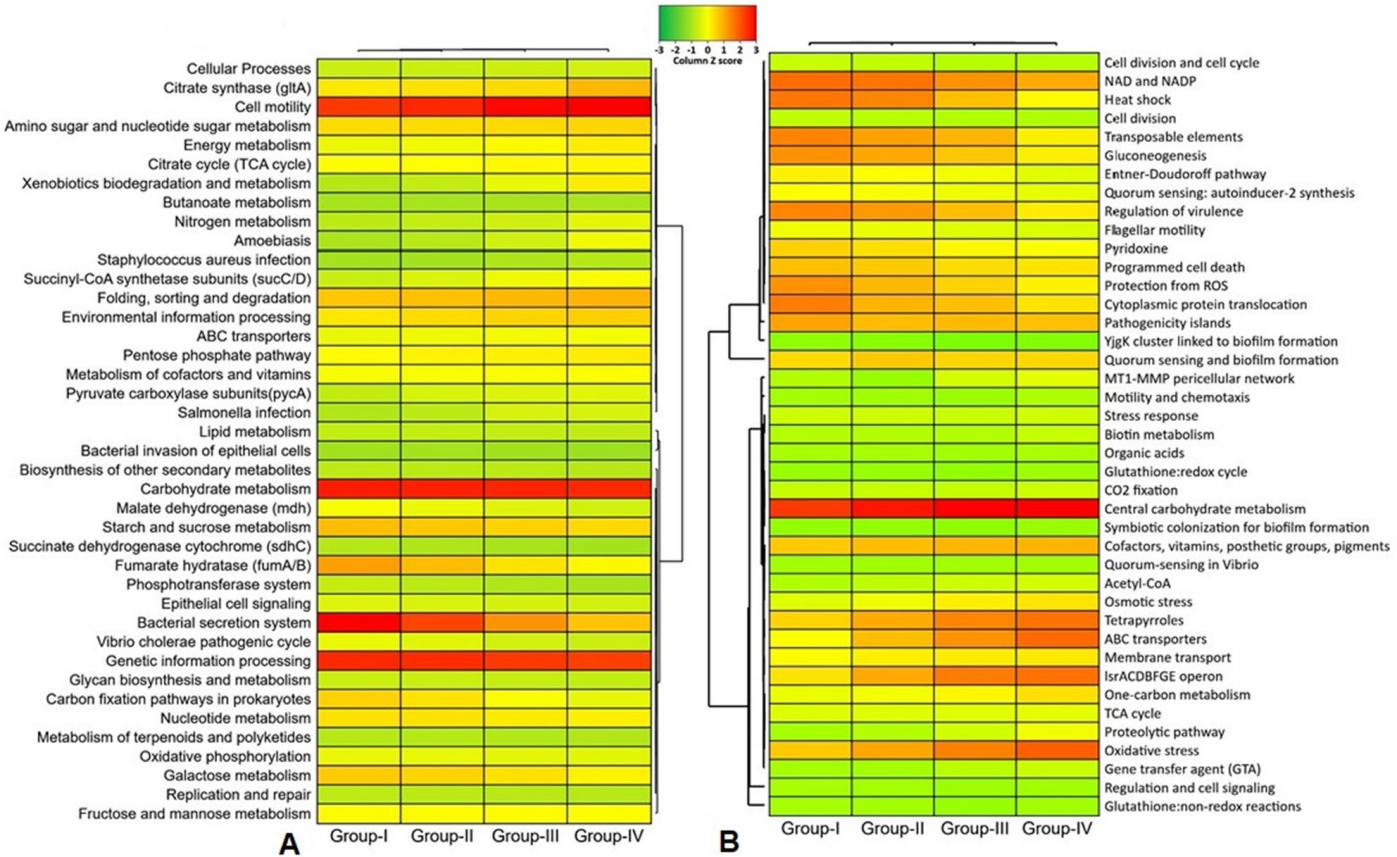
Functional genomic potentials of the anaerobic digestion associated microbial community through KEGG and SEED Pathways analysis. (**A**) Heatmap depicting the distribution of the 40 genes associated with the identified metabolic functional potentials detected by KEGG Pathways analysis within the four metagenomic groups of the AD microbiome. (**B**) Heatmap showing the distribution of the 41 functional gene composition and metabolic potential detected by SEED Pathways analysis within the four metagenomic groups of the AD microbiome. The color code indicates the presence and completeness of each gene, expressed as a value (Z score) between −2 (low abundance), and 2 (high abundance). The red color indicates the highest abundance whilst light green cells account for lower abundance of the respective genes in each metagenome. FunRich (http://funrich.org/) tool was used to these heatmaps.

**Figure 10:**
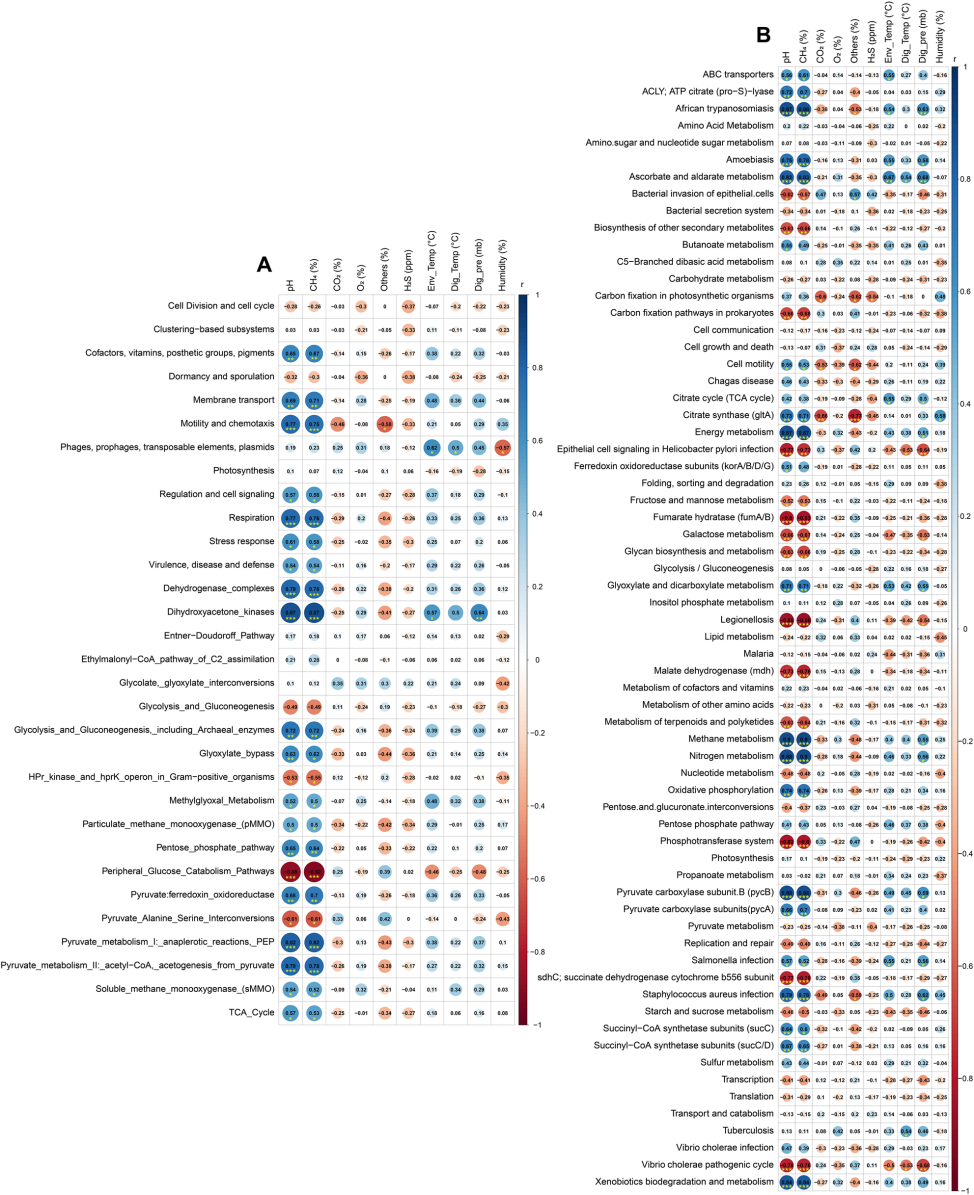
The correlation between different physicochemical parameters and genomic functional potentials of the AD microbiomes. The numbers display the Spearman’s correlation coefficient (r). Blue and red indicate positive and negative correlation, respectively. The color density, circle size, and numbers reflect the scale of correlation. *Significant level (*p < 0.05; **p < 0.01; ***p < 0.001). (**A**) Correlation analysis of different physicochemical parameters and SEED subsystems. (**B**) Correlation analysis of different physicochemical parameters and KEGG pathways. The R packages, Hmisc (https://cran.r-project.org/web/packages/Hmisc/index.html) and corrplot (https://cran.r-project.org/web/packages/corrplot/vignettes/corrplot-intro.html) were used respectively to analyze and visualize the data.

Functional metabolic profiling of the gene families of the same KEGG pathway for AD microbiomes revealed significant differences (p = 0.012, Kruskal–Wallis test) in their relative abundances, and positive correlation with level of energy production (Fig. 9, Data S2). Among the detected KO modules, genes coding for CHO metabolism and genetic information and processing were top abundant, however did not vary significantly across the metagenome groups. Remarkably, the relative abundance of genes coding for energy metabolism, xenobiotics biodegradation and metabolism, butanoate metabolism, citrate synthase (*glt*A), succinyl-CoA synthetase subunits (*sucC*/D), pyruvate carboxylase subunits (*pycA*) and nitrogen metabolism gradually increased with the increasing rate of CH_4_ production, and had several-fold over expression among the microbiomes of Group-IV. Conversely, fumarate hydratase (*fumA*/B), malate dehydrogenase (*mdh*) and bacterial secretion system associated genes were predominantly overexpressed in Group-I related microbiomes which gradually decreased with advance of digestion process, and remained more than two-fold lower expressed in the peak level of CH_4_ production (lowest in Group-IV) (Fig. 9A, Data S2). We also detected 41 statistically different (p = 0.033, Kruskal–Wallis test) SEED functions in the AD microbiomes. Overall, the top CH_4_ producing microbiomes (Group-III and Group-IV) had higher relative abundances of these SEED functions compared lower CH_4_ producing microbiomes (Group-I and Group-II), except for regulation of virulence (highest in Group-I microbes; 17.08%), gluconeogenesis (highest in Group-I microbes; 16.27%) and transposable elements (highest in Group-I microbes; 17.28%) (Fig. 9B, Data S2). The Group-IV-microbiomes (highest CH_4_ producing) were enriched in genes coding for tetrapyrroles (17.42%), one carbon (10.29%) and biotin (4.55%) metabolism, oxidative (18.76%) and osmotic (9.94%) stress, proteolytic pathway (7.74%), MT1-MMP pericellular network (6.45%), acetyl-CoA production (5.33%) and motility and chemotaxis (3.13%) compared to the microbes of the other metagenomes. The Group-I microbiomes however had a higher abundance of SEED functions involved in protection from ROS (16.28%), heat shock (18.31%) and NAD and NADP (19.03%) (Fig. 9B, Data S2). Analyzing correlation between AD physicochemical parameters and microbial genomic functions, we found that SEED subsystems such as cofactors, vitamins, prosthetic groups, pigments, membrane transport, motility and chemotaxis, respiration, stress response, dehydrogenase_complexes, dehydrogenase_ kinases, glycolysis and gluconeogenesis including archaeal enzymes, glyoxylate bypass, pentose phosphate pathway, pyruvate:ferredoxin oxidoreductase, pyruvate metabolism I and II had significant positive correlation with digester pH and CH_4_ concentration (%) (Spearman correlation; r > 0.6, p < 0.01). (Fig. 10A, Data S2). Simultaneously, the Kos like ascorbate and aldarate metabolism, citrate synthase (gltA), energy, glyoxylate, dicarboxylate, methane and nitrogen metabolism, xenobiotics biodegradation and metabolism, succinyl-CoA synthetase subunits (sucC/D) and oxidative phosphorylation were significantly positively correlated with digester pH, CH_4_ concentration (%), pressure and temperature (Spearman correlation; r > 0.6, p < 0.01). (Fig. 10B, Data S2).

## Discussion

This study is the first ever approach to reveal the dynamic shifts in microbiome composition and abundances in different levels of biogas production under the anaerobic digestion system using the state-of-the-art WMS technology along with analysis of the physicochemical parameters in Bangladesh. Anaerobic digestion of organic wastes is favored by the metabolic activities of different types of microorganisms including bacteria and archaea^30^. The physicochemical parameters assessment of the AD before and after digestion revealed that biogas production was in increasing trend up to Day 35 with periodic loading of slurry (Day 1, 2, 3, 8, 10, 14, 16, 24 and 35) at 1:1 ratio of raw CD and active sludge (Table S1). After 30 days of incubation, we found that cow wastes produced highest amount of biogas (on Day 35, CH_4_; 74.1%), and corresponds to an average pH of 7.01. Earlier studies showed that optimum pH range in an AD is 6.8 to 7.2 supporting our present findings^31^. The CH_4_ production level thereafter decrease gradually reaching 59.2% on Day 44 of processing (Fig. 1). Moreover, with the gradual increase of AD temperature and pH value (~ 7; neutral), the CO_2_ and CH_4_ concentration positively increased up to Day 35 which further correlated with the higher abundance of methanogenic bacteria. Biogas production chiefly depends on the content and chemical nature of biodegradable matter. Correlation analysis between the relative abundances of specific microbial taxa and digesters’ physicochemical parameters provides a qualitative analysis method to explore the possible roles of and the interactions among these microbes. Although, most of the dominant bacterial species had negative correlation with digester pH, CH_4_ concentration, humidity, temperature and pressure, the methanogenic archaea showed strongest positive correlation with these parameters. The biochemical parameter of cow waste (slurry) reflects the presence of high content of readily biodegradable organic matter in the first phases (up to Day 35) of anaerobic digestion^30^. The CO_2_ concentration (%) found to be varied throughout the digestion process keeping an average value of 39.52%. Our analysis revealed that OC and TN content was higher at the time of loading of slurry in the AD compared to that of highest biogas production stage (at Day 35). The amount of carbon available of the substrate determines the maximum amount of CH_4_ and CO_2_ that can be formed by anaerobic digestion^9^. Conversely, C/N ratio remained lowest in this peak stage of CH_4_ production. The OC is essential for bacterial growth, and determining of the C/N ratio is essential for optimal biogas production^32^. Moreover, the total content of phosphorus, Sulphur and heavy metals (chromium, lead and nickel) also remained lowest at this highest stage of biogas production (Day 35). The lowest chromium, lead and nickel concentration during highest CH_4_ producing stage might be associated with their higher abundances of heavy metal resistance genes, and small concentrations of these metals found in the process are essential for microbial maintenance^33,34^. Certain specific metals such as Sulphur, cobalt and nickel serve as cofactors in the enzymes involved in the formation of CH_4_ during anaerobic processing^35^. However, the minerals (e.g. zinc and copper) content of the AD did not vary throughout the digestion process revealing their important roles in various metabolic pathways of anaerobic digestion^33^. Since, CH_4_ gradually declined after Day 35 of digestion irrespective differences in other physicochemical parameters, we collected samples for microbiome analysis only up to peak level CH_4_ production (Day 35). The limitation of the study is that a complete correlation between heavy metal concentration and microbial community in terms of CH_4_ is missing, so we actually do not know the effect of the heavy metals on the single microorganism, but to perform this kind of analysis, the methods should be also changed significantly. Energy production in the AD is carried out by a series of biochemical reactions including hydrolysis, acidogenesis, and acetogenesis performed by fast-growing bacteria through which complex organic macromolecules into volatile fatty acids (VFA), CO_2_, and H_2_. However, in many cases, these reactions occur at low pH, while methanogenesis is performed by slower-growing bacteria that thrive best at neutral pH^36^. Remarkably, the pH, CH_4_ concentration, pressure and temperature were the main driving factors for variations in microbial community structure in the AD as also reported in previous studies^25,30,36^.

The within (alpha) and between (beta) sample diversity of the AD microbiomes showed that that microbial dysbiosis in the AD is closely linked to different levels of biogas production. Compared to loading phase of AD (Group-I), increased microbial diversity and species richness was observed in the later phases (Group-II, Group-III and Group-IV) of anaerobic digestion. Beta diversity also revealed a substantial microbial disparity in different levels of biogas production, and segregated the samples accordingly. Despite having higher taxonomic resolutions, the microbiomes of the AD remained inconsistent and fluctuates more in Group-II, Group-III and Group-IV than those of Group-I metagenome. The taxonomic annotations of the four groups of AD showed that they were a reservoir of bacteria, followed by archaea and viruses, which corroborated the findings of other studies^37,38^. Among the identified domains, bacteria dominated in abundance, comprising 81.80% of the total microbial populations, followed by archaea (15.43%), while viruses (2.77%) comprised the least abundant population. The observed high bacterial abundances suggest their crucial metabolic roles in biomass conversion and other reactions within the reactor systems^38^. The identified affiliates of archaea were mostly consumers of smaller substrates that were generated by the bacterial taxa. The archaeal species are able to use different methanogenic routes to convert the substrates into methane gas. Nevertheless, the main roles of the identified less abundant bacteriophages were unclear, though the strains could have been active in degrading other microbial cells in the AD systems^37,38^.

In this study, the most of the dominant methanogenic archaeal species (n = 12) were belonged to phylum *Euryarchaeota* and class *Methanomicrobia* and these findings are consistent with several previous studies^17,38^ on biogas plants where phylum *Euryarchaeota (*class *Methanomicrobia*) was reported as the dominant archaea contributing to the energy production.

The methanogenic archaea are obligate anaerobes that can reduce simple substrates to methane (CH_4_) to produce cellular energy. Most of the dominant archaeal species belonged to *Methanosarcinales* and *Methanobacteriales* orders which are syntrophic acetate oxidization digesters^39^. *M. vacuolata* can play roles in energy production by utilizing methylamines, and resistance to harsh conditions^40^. *Methanoculleus* sp. are acetate-oxidising bacteria, which convert acetate directly to methane and of considerable importance in ammonia-rich engineered biogas processes^41^. Several members of *Firmicutes* and *Bacteroidetes* phyla are well known as fermentative and syntrophic bacteria, and can ferment cellulose and cellobiose to produce acetic acid, ethanol, CO_2_/H_2_ and CH_4_^42^. Moreover, the enriched consortia of *Fibrobacteres*, *Spirochaetes*, *Actinobacteria*, *Proteobacteria*, and *Thermotogae* phyla had negative correlations with physicochemical parameters, indicating some members working on acetate and butyrate syntrophic oxidation might be dominant in the AD^42^. The *Candidatus Cloacimonetes* phylum is reported to play a role in hydrolysis of cellulose and/or fermentation of hydrolysis products in AD^43^. In this study, *Firmicutes*, *Bacteroidetes*, *Proteobacteria*, *Actinobacteria*, *Spirochaetes* and *Fibrobacteres* were the most abundant bacterial phyla, and their relative abundance also varied according to the level of energy production in the corresponding sample groups. The bacterial phyla *Firmicutes* and *Bacteroidetes* appeared to dominate biogas communities in varying abundances depending on the apparent process conditions corroborating with earlier studies^43^. The observed community composition at phylum level, with dominance of *Firmicutes, Bacteroidetes* and *Proteobacteria*, is also in line with previous findings for biogas reactors^34^. The first three phases (hydrolysis/cellulolysis, acidogenesis/fermentation and acetogenesis) of the anaerobic digestion are solely performed by fermentative Bacteria. In this study, the bacterial phyla *Firmicutes* (37.0–42.0%) and *Bacteroidetes* (22.0–38.0%) appeared to dominate biogas communities in varying abundances depending on the apparent process conditions. The initial phase of the AD digestion process involves hydrolytic reactions that convert large macromolecules into smaller substrates^37^. In addition, the nature and composition of the substrates, availability of nutrients and ammonium/ammonia contents can affect both the composition and diversity of the methanogenic archaea^37^. Only certain methanogenic archaea are able to synthesize CH_4_ from the end products of bacterial fermentation. The performance and efficiency of these processes depend to a large extent on the presence of appropriate and adequate microorganisms along with the physicochemical conditions of the digester and quality of the substrates (organic materials) etc. The degree and rate of degradation (hydrolysis, fermentation, acetogenesis, and methanogenesis) and the biogas yield depend not only on the chemical and physical characteristics of the substrates, but also on the chosen process parameter such as temperature, humidity and retention time, that shape the composition of different microbial groups and communities that active in the process^34^. During the anaerobic process *Clostridiales* and *Bacteroidales* were identified as the top abundant order in all of the four metagenome groups. The large number and proportion of members of the *Clostridiales* order are indicative of the important role in the proper functioning of the microbial community in an AD fed with complex substrates. Numerous members of the *Clostridiales* order, and members of the *Clostridiaceae* family are capable of performing diverse fermentation pathways and known to use lactate and acetate as electron sources for the reduction of iron and cobalt under anaerobic condition. They primarily ferment sugars to organic acids through the reductive acetyl-CoA pathway, which is typical in acetogenic bacteria and in some *Archaea*^4^. In this process, CO_2_ is reduced to CO and then converted to acetyl-CoA, H_2_ serving as electron donor. It should additionally be noted that a large number of *Clostridia* actively produce H_2_, an important substrate for the hydrogenotrophic methanogens^4,44^. In anaerobic environments, *Clostridiales* has been reported as the main cellulose degrader^45^, and play important role in the hydrolysis step^34^. These findings are in line with many of the previous reports^37,46^ who reported the potential roles of these bacterial taxa in the efficient and increased production of biogas under anaerobic condition^47^. In addition, bacteria belonging to the order *Bacteroidales* have been suggested to be involved in the degradation of lignocellulose materials, such as straw and hay, the chief component of cattle feed^47,48^. The identified affiliates of *Bacteroidales* belong to *Bacteroidetes*, and are majorly known to ferment carbohydrates and proteins, concomitantly releasing H_2_^33^. Moreover, *Bacteroides*, the predominating genus in the AD coming from *Bacteroidales* order were observed to co-exist with methanogenic archaea possibly to increase energy extraction from indigestible plant materials^33^, and we hypothesize that they are the key drivers of the observed *β*-diversities since the relative abundance of this genus gradually decreased with the digestion process.

The digestion process of the AD was carried out by the integrated cross-kingdom interactions since both bacteria and archaea were simultaneously detected in this WMS-based study corroborating with several earlier reports^22,23,26^. The strain-level taxonomic profiling revealed that methanogenic archaeal strains were more prevalent than bacterial strains. The archaeal domain of the AD microbiomes was composed of different strains of methanogenic, hydrogenotrophic and thermophilic genera of *Methanoculleus*, *Methanosarcina*, *Methanothrix*, *Methanobacterium* and *Methanobrevibacter* genera. The current findings are corroborated with many of the earlier studies who reported that these genera to be predominantly abundant in the AD of manures^30^, and associated with biogas production under anaerobic conditions^49^. These methanogenic genera might reside in the microenvironments appropriate for anaerobic metabolism^26^ and their presence has been reported in microbial communities producing biogas^50^. Members of *Methanoculleus* are hydrogenotrophic methanogens^51^, while *Methanosarcina* species or strains are mostly acetoclasic but also able to use H_2_^24,52^. In addition, *Methanosarcina* spp. has been reported to have higher growth rates and tolerance to pH changes and could potentially lead to stable methanogenesis in the AD^10,53^. *Methanobrevibacter* was predominant in initial phase of digestion (Day 2 and 15) in the bioreactor, and are known to be hydrogenotrophic, by using CO_2_ and H_2_ as substrates to generate biomethane^24,50^. These archaeal genera are suggested to play vital role in hydrogenotrophic methanogenesis, and maintaining methanogenic community diversity^21,25^.

We found significant association across different methanogenic species with increased level of CH_4_ production^54^, with particular emphasis in the highest CH_4_ producing metagenome (Group-IV) of the AD. Mounting evidence suggested that, under anaerobic condition, the functional composition of the energy producing microbial communities are closely related to the physicochemical and environmental factors such as pH, temperature and pressure of the digester^10,30,38,55^. The ecological functions of microorganisms living in similar environments remained more similar, but the composition of microbial species (especially, the dominant species) performing the functions differed greatly corroborating with the results of several previous studies^10,24,56^. The methanogenic archaea are obligate anaerobes that can reduce simple substrates to methane (CH_4_) to produce cellular energy. Most of the dominant archaeal species belonged to *Methanosarcinales* and *Methanobacteriales* orders which are syntrophic acetate oxidization digesters^39^. *M. vacuolata* can play roles in energy production by utilizing methylamines, and resistance to harsh conditions^40^. *Methanoculleus sp.* are acetate-oxidising bacteria, which convert acetate directly to methane and of considerable importance in ammonia-rich engineered biogas processes^41^. Several members of *Firmicutes* and *Bacteroidetes* phyla are well known as fermentative and syntrophic bacteria, and can ferment cellulose and cellobiose to produce acetic acid, ethanol, CO_2_/H_2_ and CH_4_^42^. Moreover, the enriched consortia of *Fibrobacteres*, *Spirochaetes*, *Actinobacteria*, *Proteobacteria*, and *Thermotogae* phyla had negative correlations with physicochemical parameters, indicating some members working on acetate and butyrate syntrophic oxidation might be dominant in the AD^42^. The *Candidatus Cloacimonetes* phylum is reported to play a role in hydrolysis of cellulose and/or fermentation of hydrolysis products in AD^43^.

The differences in ARGs profiles throughout different stages of anaerobic digestion were greatly affected by the taxonomic composition of microbiomes (archaea and bacteria), and the physicochemical parameters of the AD. The observed differences in microbiome composition and AD physicochemical properties are considered to be co-selection factors for antibiotic resistance^57^. Moreover, we also found that cross-resistance (where a single microbe is related to resistance to both antibiotics and metals) and coregulation (a shared regulatory system for antibiotic and metal resistance) may also lead to the increases in ARGs^55^. Therefore, to understand the mechanism that allows heavy metals to influence ARGs, the microbial community, heavy metal resistance genes, and their relationships with ARGs should be research forward. Overall, the diversity and abundance of the ARGs determined in this study were high, which clearly indicates that the unmonitored use of antibiotics and metals in dairy farms can subsequently increase the abundance of ARGs in AD systems. Our results also showed that common indicator bacteria such as *E. coli*, *Salmonella* and *Staphylococcus* species were not found in indicator species analysis, and these findings are supported by several previous reports of the absence of common indicator bacteria after 30 days digestion in the experimental AD at different temperatures (25–45 °C)^58^. Spearmen correlation analysis showed negative correlation between Group-II and Group-IV microbiomes. These findings are in line with the metabolic functional potentials of the AD microbiomes since fumarate hydratase (*fumA*/B), malate dehydrogenase (*mdh*) and bacterial secretion system associated genes were predominantly overexpressed in Group-I and Group-III microbiomes, which gradually decreased with advance of digestion process, and remained more than two-fold lower expressed in highest CH_4_ production stage (Group-IV). The microbial communities present in the early phage of digestion process (Group-I) increased both composition and abundances in the second phase of digestion (Group-II metagenome) in ambient growth conditions of the AD. With the advance of digestion time, the increasing efficiency of anaerobic digestion creates a favorable environment for the methanogens, and thus found in higher composition and abundances in Group-III and Group-IV metagenomes^21,25^. In addition, the Group-IV-microbiomes showed higher genomic functional activities related to tetrapyrroles, one carbon and biotin metabolism, oxidative and osmotic stress, proteolytic pathways, MT1-MMP pericellular network, acetyl-CoA production, and motility and chemotaxis compared to the microbes of the other groups. The energy and one-carbon metabolism are associated with certain common central pathways which are universally present across in many bacterial species, despite the wide variety of growth substrates and conditions they utilize^59^. Evidence of environmental stress conditions prevailing within the bioreactors suggested that microorganisms underwent to oxidative stress corroborating our present findings^60^. Gut microbiota has considerable proteolytic power, converting ingested dietary protein. Identified bacterial species particularly *Clostridia*, *Streptococci*, *Staphylococci*, and *Bacillus* showed higher proteolytic activity which differed both in quantity and quality of protein degradation in different stage anaerobic digestion, and these findings are in line with many earlier studies^61^. Though, these findings also support the taxonomic dysbiosis of microbiomes in Group-IV metagenome, however further comprehensive study is needed to elucidate the modulation of microbiome shifts, their functional potentials and genomic expression using a larger dataset. It is therefore imperative to carryout future research on biogas-producing microbiomes which will further help in the development and improvement of anaerobic digestion models and enhance AD efficiency and stability. Such research will benefit greatly from the continued improvement of the omics technologies, particularly metaproteomic and metametabolomics, and standardized methods of bioinformatics analyses.

## Conclusions

The level of biogas production increased gradually up to Day 35 (highest CH_4_ concentration), and declined thereafter under controlled environment of the AD. The physicochemical analysis showed significant positive correlation with the level of energy production and digester pH, O_2_ level, and environmental temperature. In the AD microbiomes, *Firmicutes*, *Bacteroidetes*, *Fibrobacteres*, *Spirochaetes*, *Actinobacteria*, *Candidatus Cloacimonetes*, *Proteobacteria*, and *Thermotogae* were the dominant bacterial phyla while *Euryarchaeota* was the only predominant archaeal phylum. The indicator species analysis revealed that microbiomes of Group-III and Group-IV, for instance, *M. vacuolate*, *D. mccartyi*, *Methanosarcina* sp. Kolksee and *M. barkeri* were highly specific species for energy production. The correlation network analysis of the indicator species showed that different strains of *Euryarcheota* and *Firmicutes* phyla were negatively correlated but associated with highest level of energy production. The AD microbiomes harbored different ARGs of which genes coding for tetracyclines (doxycycline and tetracycline), macrolides (erythromycin and streptogramin B) and beta-lactams resistance remained over expressed. Additionally, the physicochemical properties of the digester including pH, CH_4_ concentration (%), pressure, temperature and environmental temperature were found to be positively correlated with these genomic functional potentials and distribution of ARGs and metal resistance pathways. Taken together, a high-level abundance of the dominant and indicator microbial communities, associated genomic functional potentials, and their correlation with the physicochemical parameters of the AD were found to be driving factors for high proportion of the energy production.

## Methods

### Digester setup and experiment design

The experiment was conducted using an AD plant prepared with provisions to measure temperature, slurry and gas sample collection and substrate charging. The biogas plant consisted of a digester, inlet-chamber, three slurry outlet pipes, gas outlet pipe and thermometer (Fig. S1). The volume of the AD was 3000 L and made of flexible polyvinyl chloride (PVC) fabric with thickness: 1.2 Mm. There was a specialized ball valve which ensures the anaerobic condition within the digester and control the flow of substrates. The temperature of the AD was monitored using a probe. The experiment was conducted for 45 days (Day 0 to Day 44). The AD was loaded 12 times throughout the experiment period with a total of 1,087 kg raw CD (highest input volume = 375 kg and lowest input volume = 35 kg) (Table S1). Initially, the digester was started with the highest loading dose of 375 kg feedstock where the ratio of raw CD and active sludge was 1:1. The raw semi-solid CD was mixed with seed sludge from previous biogas plant (slurry) before charged into the AD. The AD was portable, light in weight, low-priced and retained more heat inside.

### Sample collection and physicochemical parameters analysis

The representative samples (n = 16) including CD, slurry and active sludge (AS) were collected and stored for subsequent analysis. The samples were categorized into four groups (Group-I, Group-II, Group-III and Group-IV) based on collection time (Day 0 to Day 35) and level of energy production (CH_4_%). The samples of Group-I (n = 2) were collected at Day 0 (day of first input) when the CH_4_ concentration of the digesta was 0.0% with an average pH of 5.44. Likewise, Group-II samples (n = 5) were collected at Day 2 and Day 7 of the digestion process when the CH_4_ concentration and pH of the digesta were 21 and 5.44%, and 34 and 5.56%, respectively. The sampling of the Group-III (n = 5) was done at Day 10 and Day 27 of the digestion process having the CH_4_ concentration and pH of the digesta 47.4 and 5.97%, and 58.2 and 6.87%, respectively. The Group-IV included samples (n = 4) collected at Day 34 and Day 35 of digestion when the CH_4_ concentration and pH of the digesta were 71.4 and 6.99%, and 74.1 and 7.01%, respectively (Table 1). Therefore, highest CH_4_ production (74.1%) was recorded at Day 35 of the digestion process. Thereafter, the CH_4_ concentration gradually declined (up to Day 44), and thus, no samples were collected during this stage (Day 36–Day 44) for metagenomic analysis. The TN content was measured by micro-Kjeldahl method^62^ while phosphorus, potassium, heavy metals (Lead, Zinc, Nickel, Cadmium, Chromium), OC, Sulphur and moisture content were determined by spectrophotometric molybdovanadate^63^, flame photometric^64^, atomic absorption spectrophotometric^63^, wet oxidation, turbidimetric and gravimetric methods from the Department of Soil Science, University of Dhaka. The detection limit of metals was of the order of 0.1 μg L^−1^ ^30^.

### Metagenomic DNA extraction, sequencing and bioinformatics analysis

We extracted the total genomic DNA from 16 samples (Data S1) using an automated DNA extraction platform with DNeasy PowerSoil Kit (QIAGEN, Germany) according to manufacturer’s instructions. Extracted gDNA was quantified and purity checked through NanoDrop (ThermoFisher, USA) with an absorbance ratio of 260/280. Shotgun metagenomic (WMS) libraries were prepared with Nextera XT DNA Library Preparation Kit (Hoque et al., 2019), and paired-end (2 × 150 bp) sequencing was performed on a NovaSeq 6000 sequencer (Illumina Inc., USA) from the Macrogen Inc. (www.macrogen.com) Seoul, Republic of South Korea. The gDNA from sixteen samples generated 380.04 million reads, and the average reads per sample was 23.75 million (maximum = 24.79 million, minimum = 20.75 million) (Data S1). The low-quality reads from the generated FASTQ files were filtered and removed through BBDuk (with options k = 21, mink = 6, ktrim = r, ftm = 5, qtrim = rl, trimq = 20, minlen = 30, overwrite = true) (Stewart et al. 2018). In this study, on an average 21.45 million reads per sample (maximum = 23.75 million, minimum = 18.85 million) passed the quality control step (Data S1).

### Microbiome analysis and AMR profiling

The taxonomic assignment of the generated WMS data was performed using both mapping based and open-source assembly-based hybrid methods of PahtoScope 2,0 (PS)^65^ and MG-RAST 4.0 (MR)^66^. In PS analysis, the NCBI Reference Sequence Database (NCBI RefSeq Release 201) for bacteria and archaea was prepared by Kraken2^67^. The reads were then aligned against the target (RefSeq) libraries using Minimap2^68^, and filtered to remove the reads aligned with the cattle genome (bosTau8) and human genome (hg38) using BWA^69^ and samtools^70^. In PS analysis, we employed the PathoID module to get exact read counts for downstream analysis^65^. We simultaneously uploaded the raw sequences to the MR server with proper metadata. In MR analysis, the uploaded raw reads were subjected to optional quality filtering with dereplication and host DNA removal, and finally annotated for taxonomic assignment. Less than 100 hits from PS taxonomy were filtered for the downstream analysis. Read normalization in each sample was performed using median sequencing depth through Phyloseq (version 4.0) package in R. The within sample (alpha) diversity of microbial communities was calculated using the Shannon and Simpson diversity indices^71^ through the “Vegan” package in R. To evaluate alpha diversity in different groups, we performed the non-parametric test Kruskal–Wallis rank-sum test. The diversity across the sample groups (Beta-diversity) was measured with the principal coordinate analysis (PCoA) using Bray–Curtis dissimilarity matrices, and permutational multivariate analysis of variance (PERMANOVA) with 999 permutations to estimate a p-value for differences among groups^72^. Phyloseq and Vegan packages were employed for these statistical analyses^73^. Dominant microbial community were also determined where ≥ 1% read in at least one samples were considered. After filtering there were 43 taxa remained in 16 samples. Spearman’s correlation analyses between dominant microbiotas and physicochemical parameters were performed in rcorr function of Hmisc^74^ and corrplot function of corrplot^75^ R package.

Indicator species specific to a given sample group (having ≥ 1000 reads assigned to a taxon) were identified based on the normalized abundances of species using the R package indicspecies^76^, and the significant indicator value (IV) index was calculated by the 999-permutation test. Larger IV indicates greater specificity of taxa and p < 0.05 was considered statistically significant^76^. Network analysis was used to explore the co-occurrence patterns of the energy producing bacterial and archaeal taxa across the metagenome groups. In addition, the Spearman’s correlation coefficient and significance tests were performed using the R package Hmisc. A correlation network was constructed and visualized with Gephi (ver. 0.9.2). We detected the antibiotics resistance genes (ARGs) among the microbiomes of four metagenomes through the ResFinder 4.0 database (https://bitbucket.org/genomicepidemiology/resfinder/src/master/). The ResFinder database was integrated within AMR++ pipeline (https://megares.meglab.org/amrplusplus/latest/html/v2/)^77^ to identify the respective antimicrobial resistance genes and/or protein families^26^.

### Functional profiling of the microbiomes

In addition to taxonomic annotations, the WMS reads were also mapped onto the Kyoto Encyclopedia of Genes and Genomes (KEGG) database^78^, and SEED subsystem identifiers, respectively, on the MR server^66^ for metabolic functional profiling. The functional mapping was performed with the partially modified set parameters (*e-*value cutoff: 1 × 10^−30^, min. % identity cutoff: 60%, and min. alignment length cutoff: 20) of the MR server^26^.

### Statistical analysis

To evaluate differences in the relative percent abundance of taxa in AD (Group-I, Group-II, Group-III and Group-IV) for PS data, we used the non-parametric test Kruskal–Wallis rank sum test. Pairwise Pearson’s correlation was performed to reveal the association between each of the two physicochemical parameters of the AD and microbial alpha diversity characteristics. We normalized the gene counts by dividing the number of gene hits to individual taxa/function by total number of gene hits in each metagenome dataset to remove bias due to differences in sequencing efforts. The non-parametric test Kruskal–Wallis rank sum tests were also performed to identify the differentially abundant SEED or KEGG functions (at different levels), and antimicrobial resistance (ARGs) in four metagenomes. All the statistical tests were carried out using IBM SPSS (SPSS, Version 23.0, IBM Corp., NY USA). To calculate the significance of variability patterns of the microbiomes (generated between sample categories), we performed PERMANOVA (Wilcoxon rank sum test using vegan 2.5.1 package of R 3.4.2) on all four sample types at the same time and compared them pairwise. A significance level of alpha = 0.05 was used for all tests. Spearman’s correlation of dominant microbial community, KEGG pathways and SEED subsystem functional data to physical parameters of the AD were performed in Hmisc^74^ and corrplot^75^ R packages as described above.

## Supplementary Information

Supplementary Information 1.

Supplementary Information 2.

Supplementary Figure 1.

Supplementary Figure 2.

Supplementary Figure 3.

Supplementary Figure 4.

Supplementary Figure 5.

Supplementary Figure 6.

Supplementary Figure 7.

Supplementary Figure 8.

Supplementary Table S1.

Supplementary Table S2.

## Data Availability

The sequence data reported in this article have also been deposited in the National Center for Biotechnology Information (NCBI) under BioProject accession number PRJNA668799. Supplementary materials supporting the results of the study are available in this article as Data S1 and S2, Figs. S1-S8, and Table S1.

## References

[1] Khan EU, Mainali B, Martin A, Silveira S (2014). Techno-economic analysis of small scale biogas based polygeneration systems: Bangladesh case study. Sustain. Energy Technol..

[2] Rahman KM, Melville L, Edwards DJ, Fulford D, Thwala WD (2019). Determination of the potential impact of domestic anaerobic digester systems: A community based research initiative in Rural Bangladesh. Processes.

[3] Weiland P (2010). Biogas production: current state and perspectives. Appl. Microbiol. Biotechnol..

[4] Wirth R (2012). Characterization of a biogas-producing microbial community by short-read next generation DNA sequencing. Biotechnol. Biofuels.

[5] Gupta KK, Aneja KR, Rana D (2016). Current status of cow dung as a bioresource for sustainable development. Bioresour. Bioprocess..

[6] Randhawa, G. K. & Kullar, J. S. Bioremediation of pharmaceuticals, pesticides, and petrochemicals with gomeya/cow dung. *Int. Sch. Res. Notices* **2011** (2011). 10.5402/2011/362459 PMC3197002 22084712

[7] Umanu G, Nwachukwu S, Olasode O (2013). Effects of cow dung on microbial degradation of motor oil in lagoon water. GJBB.

[8] Das A, Sahoo S, Rana S (2018). Sustainable conservation of kitchen wastes into fuels and organic fertilizer. Int. J. Eng. Sci. Technol..

[9] Mulka R, Szulczewski W, Szlachta J, Prask H (2016). The influence of carbon content in the mixture of substrates on methane production. Clean Technol. Environ. Policy.

[10] Tian Z, Cabrol L, Ruiz-Filippi G, Pullammanappallil P (2014). Microbial ecology in anaerobic digestion at agitated and non-agitated conditions. PLoS ONE.

[11] Campanaro S (2020). New insights from the biogas microbiome by comprehensive genome-resolved metagenomics of nearly 1600 species originating from multiple anaerobic digesters. Biotechnol. Biofuels.

[12] Valentinuzzi F (2020). The fertilising potential of manure-based biogas fermentation residues: Pelleted vs. liquid digestate. Heliyon.

[13] Fernandez-Gonzalez N, Braz G, Regueiro L, Lema J, Carballa M (2021). Microbial invasions in sludge anaerobic digesters. Appl. Microbiol. Biotechnol..

[14] Theuerl S, Klang J, Hülsemann B, Mächtig T, Hassa J (2020). Microbiome diversity and community-level change points within manure-based small biogas plants. Microorganisms.

[15] Ziels RM (2018). Microbial rRNA gene expression and co-occurrence profiles associate with biokinetics and elemental composition in full-scale anaerobic digesters. Microbial Biotechnol..

[16] Mukti RF, Sinthee SS (2019). Metagenomic approach: transforming in-silico research for improved biogas production. Int. J. Res. Appl. Sci. Biotechnol..

[17] Bremges A (2015). Deeply sequenced metagenome and metatranscriptome of a biogas-producing microbial community from an agricultural production-scale biogas plant. Gigascience.

[18] Ziganshin AM, Ziganshina EE, Kleinsteuber S, Nikolausz M (2016). Comparative analysis of methanogenic communities in different laboratory-scale anaerobic digesters. Archaea.

[19] Hoque M, Das Z, Rahman A, Haider M, Islam M (2018). Molecular characterization of *Staphylococcus aureus* strains in bovine mastitis milk in Bangladesh. Int. J. Vet. Sci. Med..

[20] Saha O (2020). Multidrug-resistant avian pathogenic *Escherichia coli* strains and association of their virulence genes in Bangladesh. Microorganisms.

[21] Zhang Q (2019). High variations of methanogenic microorganisms drive full-scale anaerobic digestion process. Environ. Int..

[22] Hoque MN (2020). Insights into the resistome of bovine clinical mastitis microbiome, a key factor in disease complication. Front. Microbiol..

[23] Hoque MN (2019). Metagenomic deep sequencing reveals association of microbiome signature with functional biases in bovine mastitis. Sci. Rep..

[24] Zhu X (2020). Metabolic dependencies govern microbial syntrophies during methanogenesis in an anaerobic digestion ecosystem. Microbiome.

[25] De Vrieze J (2017). Microbial community redundancy in anaerobic digestion drives process recovery after salinity exposure. Water Res..

[26] Hoque MN (2020). Microbiome dynamics and genomic determinants of bovine mastitis. Genomics.

[27] Pham JV (2019). A review of the microbial production of bioactive natural products and biologics. Front. Microbiol..

[28] Sundberg C (2013). 454 pyrosequencing analyses of bacterial and archaeal richness in 21 full-scale biogas digesters. FEMS Microbiol. Ecol.

[29] Hahnke S, Langer T, Klocke M (2018). *Proteiniborus indolifex* sp. nov., isolated from a thermophilic industrial-scale biogas plant. Int. J. Syst. Evol..

[30] El Asri O, Afilal ME, Laiche H, Elfarh A (2020). Evaluation of physicochemical, microbiological, and energetic characteristics of four agricultural wastes for use in the production of green energy in Moroccan farms. Chem. Biol. Technol. Agric..

[31] Cioabla AE, Ionel I, Dumitrel G-A, Popescu F (2012). Comparative study on factors affecting anaerobic digestion of agricultural vegetal residues. Biotechnol. Biofuels.

[32] Tanimu MI, Ghazi TIM, Harun MR, Idris A (2015). Effects of feedstock carbon to nitrogen ratio and organic loading on foaming potential in mesophilic food waste anaerobic digestion. Appl. Microbiol. Biotechnol..

[33] Agustini CB, da Costa M, Gutterres M (2020). Biogas from tannery solid waste anaerobic digestion is driven by the association of the bacterial order bacteroidales and archaeal family methanosaetaceae. Appl. Biochem. Biotechnol..

[34] Liu T, Sun L, Müller B, Schnürer A (2017). Importance of inoculum source and initial community structure for biogas production from agricultural substrates. Bioresource Technol..

[35] Zandvoort MH, van Hullebusch ED, Gieteling J, Lens PN (2006). Granular sludge in full-scale anaerobic bioreactors: Trace element content and deficiencies. Enzyme Microb. Technol..

[36] Zobeashia, S. S. L.-T., Abioye, P. O., Ijah, U. J. J. & Oyewole, O. A. The impact of physicochemical parameter in anaerobic digestion of organic wastes. *Res. Square* (2021).

[37] Muturi SM (2021). Metagenomics survey unravels diversity of biogas microbiomes with potential to enhance productivity in Kenya. PLoS ONE.

[38] Stolze Y (2015). Comparative metagenomics of biogas-producing microbial communities from production-scale biogas plants operating under wet or dry fermentation conditions. Biotechnol. Biofuels.

[39] Gao M, Guo B, Zhang L, Zhang Y, Liu Y (2019). Microbial community dynamics in anaerobic digesters treating conventional and vacuum toilet flushed blackwater. Water Res..

[40] Mosbæk F (2016). Identification of syntrophic acetate-oxidizing bacteria in anaerobic digesters by combined protein-based stable isotope probing and metagenomics. ISME J..

[41] Manzoor S, Schnürer A, Bongcam-Rudloff E, Müller B (2016). Complete genome sequence of *Methanoculleus bourgensis* strain MAB1, the syntrophic partner of mesophilic acetate-oxidising bacteria (SAOB). Stand. Genom. Sci..

[42] Jiang Y (2019). Exploring the roles of and interactions among microbes in dry co-digestion of food waste and pig manure using high-throughput 16S rRNA gene amplicon sequencing. Biotechnol. Biofuels.

[43] Limam RD (2014). Members of the uncultured bacterial candidate division WWE 1 are implicated in anaerobic digestion of cellulose. MicrobiologyOpen.

[44] Oelgeschläger E, Rother M (2008). Carbon monoxide-dependent energy metabolism in anaerobic bacteria and archaea. Arch. Microbiol..

[45] Lynd LR, Weimer PJ, Van Zyl WH, Pretorius IS (2002). Microbial cellulose utilization: fundamentals and biotechnology. Microbiol. Mol. Biol. Rev..

[46] Luo G, Fotidis IA, Angelidaki I (2016). Comparative analysis of taxonomic, functional, and metabolic patterns of microbiomes from 14 full-scale biogas reactors by metagenomic sequencing and radioisotopic analysis. Biotechnol. Biofuels.

[47] Sun L, Pope PB, Eijsink VG, Schnürer A (2015). Characterization of microbial community structure during continuous anaerobic digestion of straw and cow manure. Microb. Biotechnol..

[48] Hanreich A (2013). Metagenome and metaproteome analyses of microbial communities in mesophilic biogas-producing anaerobic batch fermentations indicate concerted plant carbohydrate degradation. Syst. Appl. Microbiol..

[49] Kouzuma A (2017). Non-autotrophic methanogens dominate in anaerobic digesters. Sci. Rep..

[50] Poehlein A, Schneider D, Soh M, Daniel R, Seedorf H (2018). Comparative genomic analysis of members of the genera Methanosphaera and Methanobrevibacter reveals distinct clades with specific potential metabolic functions. Archaea.

[51] Shcherbakova V (2011). *Methanobacterium arcticum* sp. nov., a methanogenic archaeon from Holocene Arctic permafrost. Int. J. Syst. Evol. Microbiol..

[52] Kor-Bicakci G, Ubay-Cokgor E, Eskicioglu C (2020). Comparative analysis of bacterial and archaeal community structure in microwave pretreated thermophilic and mesophilic anaerobic digesters utilizing mixed sludge under organic overloading. Water.

[53] Cho S-K (2013). Dry anaerobic digestion of food waste under mesophilic conditions: Performance and methanogenic community analysis. Bioresour. Technol..

[54] Deng S (2019). A plant growth-promoting microbial soil amendment dynamically alters the strawberry root bacterial microbiome. Sci. Rep..

[55] Mitchell SM, Ullman JL, Teel AL, Watts RJ, Frear C (2013). The effects of the antibiotics ampicillin, florfenicol, sulfamethazine, and tylosin on biogas production and their degradation efficiency during anaerobic digestion. Bioresour. Technol..

[56] Wang C-Y (2019). Soil pH is the primary factor driving the distribution and function of microorganisms in farmland soils in northeastern China. Ann. Microbiol..

[57] Andersson DI, Hughes D (2010). Antibiotic resistance and its cost: is it possible to reverse resistance?. Nat. Rev. Microbiol..

[58] Dinova N, Belouhova M, Schneider I, Rangelov J, Topalova Y (2018). Control of biogas production process by enzymatic and fluorescent image analysis. Biotechnol. Biotechnol. Equip..

[59] Chubukov V, Gerosa L, Kochanowski K, Sauer U (2014). Coordination of microbial metabolism. Nat. Rev. Microbiol..

[60] Joyce A (2018). Linking microbial community structure and function during the acidified anaerobic digestion of grass. Front. Microbiol..

[61] Rowland I (2018). Gut microbiota functions: metabolism of nutrients and other food components. Eur. J. Nutr..

[62] Sparks DL, Page A, Helmke P, Loeppert RH (2020). Methods of Soil Analysis, Part 3: Chemical Methods.

[63] Afilal M, Elasri O, Merzak Z (2014). Caractérisations des déchets organiques et évaluation du potentiel Biogaz (Organic waste characterization and evaluation of its potential biogas). J. Mater. Environ. Sci.

[64] Banerjee P, Prasad B (2020). Determination of concentration of total sodium and potassium in surface and ground water using a flame photometer. Appl. Water Sci..

[65] Hong C (2014). PathoScope 2.0: a complete computational framework for strain identification in environmental or clinical sequencing samples. Microbiome.

[66] Glass EM, Wilkening J, Wilke A, Antonopoulos D, Meyer F (2010). Using the metagenomics RAST server (MG-RAST) for analyzing shotgun metagenomes. Cold Spring Harb. Protoc..

[67] Wood DE, Lu J, Langmead B (2019). Improved metagenomic analysis with Kraken 2. Genome Biol..

[68] Li H (2018). Minimap2: pairwise alignment for nucleotide sequences. Bioinformatics.

[69] Li H, Durbin R (2010). Fast and accurate long-read alignment with Burrows-Wheeler transform. Bioinformatics.

[70] Li H (2009). The sequence alignment/map format and SAMtools. Bioinformatics.

[71] Koh H (2018). An adaptive microbiome α-diversity-based association analysis method. Sci. Rep..

[72] Beck J, Holloway JD, Schwanghart W (2013). Undersampling and the measurement of beta diversity. Methods Ecol. Evol..

[73] McMurdie PJ, Holmes S (2013). phyloseq: an R package for reproducible interactive analysis and graphics of microbiome census data. PLoS ONE.

[74] Harrell Jr, F. E. & Harrell Jr, M. F. E. Package ‘hmisc’. *CRAN2018* **2019**, 235–236 (2019).

[75] Wei T (2017). Package ‘corrplot’. Statistician.

[76] De Cáceres M, Legendre P, Wiser SK, Brotons L (2012). Using species combinations in indicator value analyses. Methods Ecol. Evol..

[77] Doster E (2020). MEGARes 2.0: a database for classification of antimicrobial drug, biocide and metal resistance determinants in metagenomic sequence data. Nucleic Acids Res..

[78] Kanehisa M, Sato Y, Furumichi M, Morishima K, Tanabe M (2019). New approach for understanding genome variations in KEGG. Nucleic Acids Res..

