# Supplementary figures and images for: Microbiome signature and diversity regulates the level of energy production under anaerobic condition
Source: Sci Rep. 2021 Oct 5;11:19777. doi: 10.1038/s41598-021-99104-3 (PMC8492712; doi:10.1038/s41598-021-99104-3)

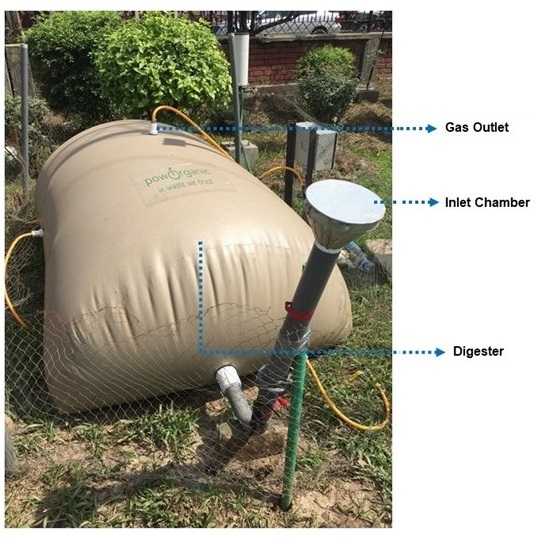

Supplement: Supplementary file 3 — Supplementary Figure 1. [file 41598_2021_99104_MOESM3_ESM.jpg]

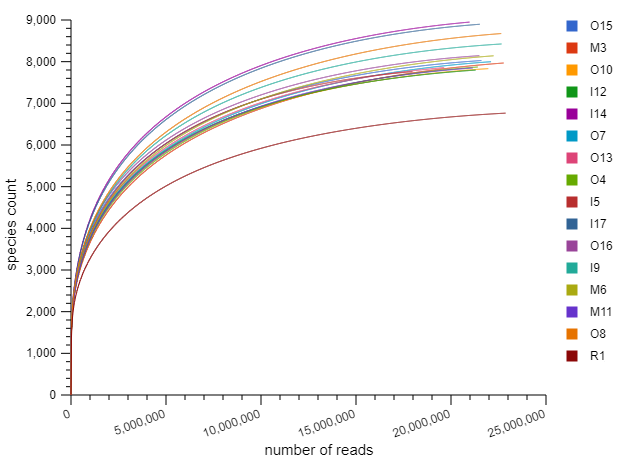

Supplement: Supplementary file 4 — Supplementary Figure 2. [file 41598_2021_99104_MOESM4_ESM.png]

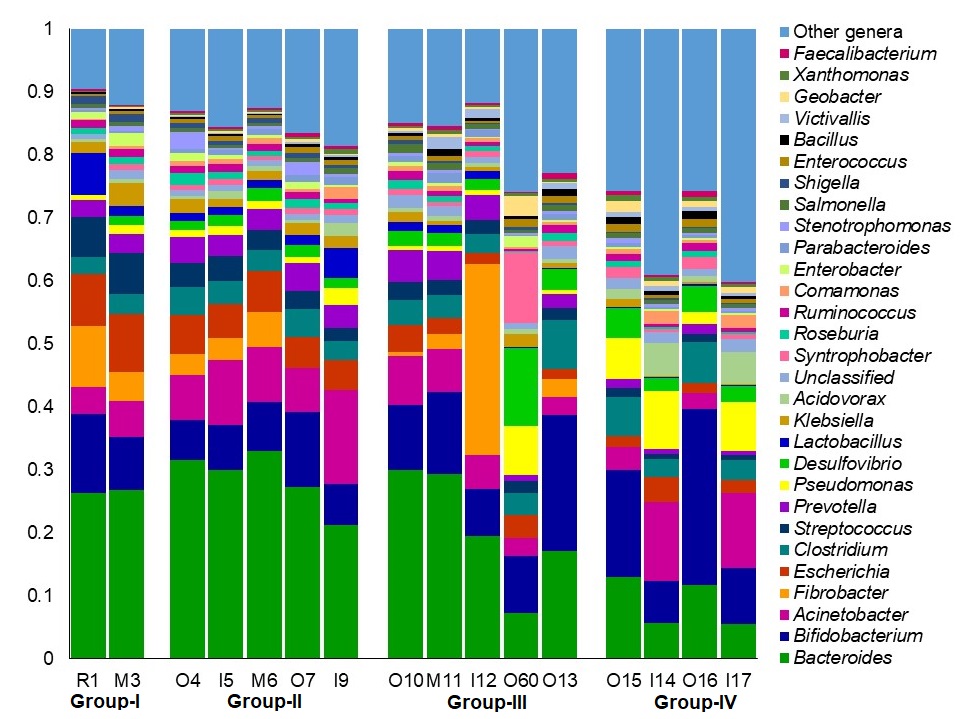

Supplement: Supplementary file 5 — Supplementary Figure 3. [file 41598_2021_99104_MOESM5_ESM.jpg]

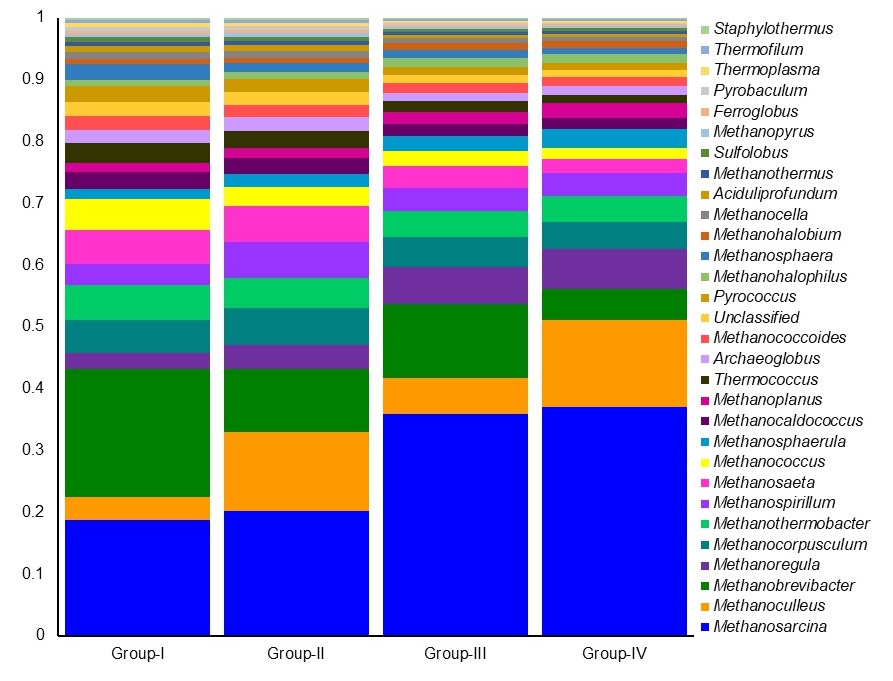

Supplement: Supplementary file 6 — Supplementary Figure 4. [file 41598_2021_99104_MOESM6_ESM.jpg]

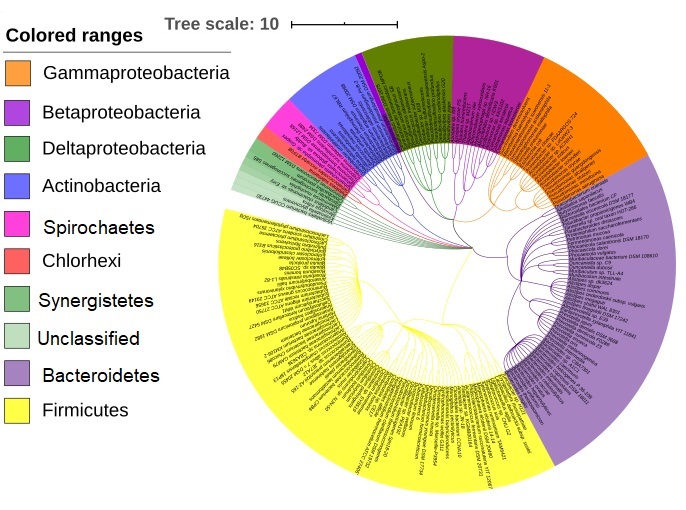

Supplement: Supplementary file 7 — Supplementary Figure 5. [file 41598_2021_99104_MOESM7_ESM.jpg]

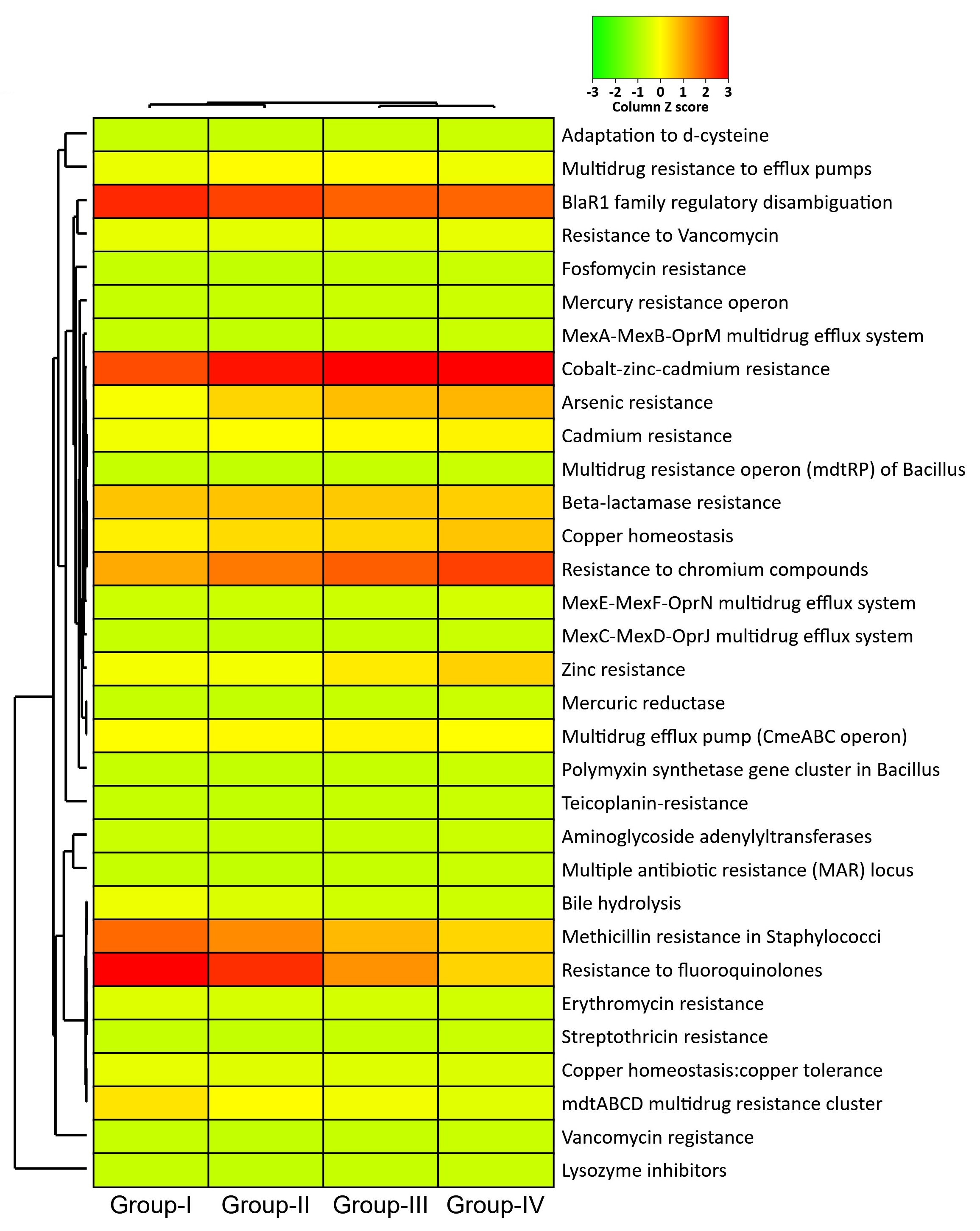

Supplement: Supplementary file 10 — Supplementary Figure 8. [file 41598_2021_99104_MOESM10_ESM.jpeg]
