# Supplementary material for: Microbiome signature and diversity regulates the level of energy production under anaerobic condition
Source: Sci Rep. 2021 Oct 5;11:19777. doi: 10.1038/s41598-021-99104-3 (PMC8492712; doi:10.1038/s41598-021-99104-3)
Supplement: Supplementary file 11 — Supplementary Table S1. [file 41598_2021_99104_MOESM11_ESM.docx]

**Table S1:** Data on cow dung and slurry added into the AD.

| SI No. | Day | Cow dung charge (Kg) |
| --- | --- | --- |
| 1 | 0 | 375 |
| 2 | 1 | 202 |
| 3 | 2 | 45 |
| 4 | 8 | 52 |
| 5 | 10 | 52 |
| 6 | 14 | 52 |
| 7 | 16 | 52 |
| 8 | 24 | 52 |
| 9 | 35 | 75 |
| 10 | 37 | 60 |
| 11 | 41 | 35 |
| 12 | 44 | 35 |
| **Average** | | **90.56** |
