# Supplementary material for: Microbiome signature and diversity regulates the level of energy production under anaerobic condition
Source: Sci Rep. 2021 Oct 5;11:19777. doi: 10.1038/s41598-021-99104-3 (PMC8492712; doi:10.1038/s41598-021-99104-3)
Supplement: Supplementary file 12 — Supplementary Table S2. [file 41598_2021_99104_MOESM12_ESM.docx]

**Table S2:** Data on the physicochemical parameters of the AD.

| Day | CH4 (%) | CO2 (%) | O2 (%) | Other (%) | H2S (ppm) | Environmental Temperature (˚C) | Digester Temperature (˚C) | Digester pressure (mb) | Humidity (%) |
| --- | --- | --- | --- | --- | --- | --- | --- | --- | --- |
| 1 | 0 | 0 | 0 | 0 | 0 | 31.8 | 29 | 0 | 94 |
| 2 | 21.9 | 54 | 0.2 | 23.9 | 560 | 33.6 | 31.3 | 0 | 46 |
| 3 | 25.6 | 56 | 0.2 | 18.1 | 938 | 35.1 | 46.2 | 37 | 48 |
| 4 | 27.9 | 55.4 | 0.2 | 16.5 | 523 | 34.5 | 46.3 | 8.18 | 72 |
| 7 | 34 | 51.4 | 0.3 | 14.4 | 72 | 35.3 | 44.1 | 12.51 | 42 |
| 8 | 36.3 | 49.2 | 0.2 | 14.2 | 95 | 34 | 41.2 | 14.42 | 42 |
| 10 | 47.4 | 43.2 | 0.3 | 9.1 | 115 | 36.8 | 51 | 56.41 | 48 |
| 11 | 49 | 42.5 | 0.2 | 8.3 | 140 | 27.9 | 31.3 | 6 | 79 |
| 16 | 50 | 42.2 | 0.3 | 7.4 | 164 | 32.5 | 41.2 | 11.56 | 65 |
| 17 | 51.3 | 41.7 | 0.3 | 6.7 | 124 | 28.1 | 43 | 18.56 | 79 |
| 18 | 54 | 39.7 | 0.6 | 5.7 | 24 | 32.3 | 40.2 | 12.26 | 54 |
| 21 | 52.3 | 41 | 0.3 | 6.5 | 131 | 37 | 38 | 7.68 | 51 |
| 22 | 53.1 | 40.2 | 0.3 | 6.4 | 192 | 35 | 38.5 | 17.06 | 50 |
| 23 | 54.3 | 39.1 | 0.4 | 6.3 | 72 | 32.3 | 38.3 | 11.25 | 48 |
| 24 | 55.6 | 37.6 | 1.1 | 5.7 | 18 | 36.9 | 31.8 | 19.35 | 77 |
| 27 | 58.2 | 37 | 0.3 | 4.3 | 167 | 32 | 33.8 | 6.38 | 60 |
| 34 | 71.4 | 29.8 | 0.2 | 0 | 8 | 35.3 | 41.2 | 15.69 | 53 |
| 35 | 74.1 | 27.7 | 0.2 | 0 | 5 | 36.8 | 33.1 | 14 | 54 |
| 36 | 61.6 | 35 | 0.3 | 3.1 | 63 | 36.3 | 37.1 | 11.12 | 53 |
| 37 | 61.5 | 34.8 | 0.4 | 3.3 | 9 | 36.1 | 36 | 14 | 58 |
| 38 | 60 | 35.9 | 0.3 | 3.8 | 194 | 36 | 35.1 | 16.44 | 59 |
| 44 | 59.2 | 36 | 0.2 | 4.6 | 117 | 35.8 | 36 | 7.61 | 51 |
